# Preparation and Characterization of Oxalyl Dicyanide

**DOI:** 10.1021/acs.inorgchem.5c05620

**Published:** 2026-02-20

**Authors:** Sven Ringelband, Theodore Cohen, Frank Tambornino

**Affiliations:** Department of Chemistry, Philipps-University Marburg, Marburg 35043, Germany

## Abstract

Oxalyl dicyanide and its 1,4-dioxane adduct were obtained as colorless crystalline solids from the reaction of oxalyl chloride with trimethylsilyl cyanide. Their crystal structures and vibrational spectra, supported by quantum chemical calculations, are presented. In the solid state, oxalyl dicyanide adopts *C*_2*h*_ symmetry with the preferred *trans*-*anti* configuration. The ^13^C NMR spectrum shows two distinct resonances at 159.8 and 109.9 ppm, while characteristic ν­(C≡N) and ν­(C=O) stretching vibrations are observed at 2243 and 1734 cm^–1^, respectively. Hydrolysis proceeds rapidly to oxalic acid and hydrogen cyanide, with transient formation of the unusual tetraol 2,2,3,3-tetrahydroxysuccinonitrile, as confirmed by single-crystal X-ray diffraction.

## Graphical abstract

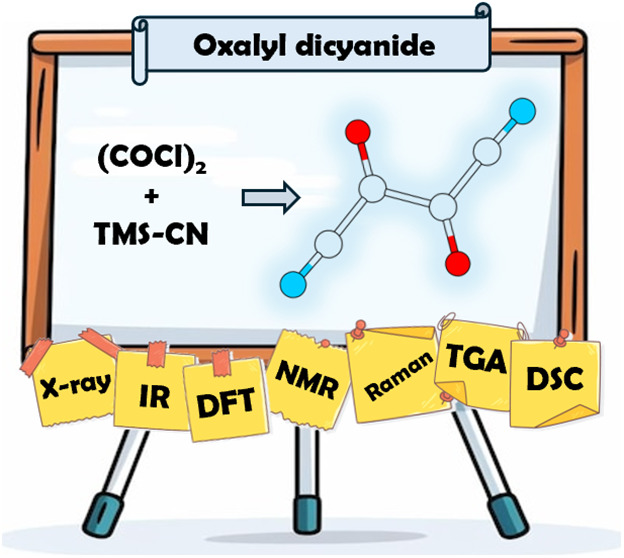

## Introduction

The history of oxalyl dipseudohalides dates back to 1898, when oxalyl diazide was first described by Curtius and Burkhardt. At that time, an aqueous solution of hydrochloric oxalyl dihydrazide was reacted with sodium nitrite to give oxalyl diazide.^1,2^ However, it was later shown to exist only as a transient intermediate under the given conditions, undergoing a Curtius rearrangement to form carbonyl amide azide upon hydrolysis. More than 60 years passed before oxalyl diazide was revisited, isolated, and its vibrational data evaluated.^3^ Since then, oxalyl pseudohalides have received little attention, with only a handful of reports published, and their chemistry remains scarcely explored. Their use as synthons is likely hampered by poor stability toward elevated temperature and moisture. In 1972, the synthesis of oxalyl dicyanide was reported, followed ten years later by oxalyl diisothiocyanate.^4,5^ More recently, detailed structural and spectroscopic data have been published for oxalyl diazide^6^ and oxalyl diisothiocyanate,^7,8^ highlighting the challenges in handling these compounds.

Due to their high reactivity, oxalyl pseudohalides may serve as model precursors for introducing functionalized carbonyl moieties into larger, more complex molecules. For related carbonyl dipseudohalides, reactivity has been shown to depend strongly on both the pseudohalide and the chalcogen atom of the carbonyl moiety. Consequently, nucleophilic reactions yield diverse products, ranging from single and double addition to intramolecular cyclization.^9,10^

Only one reference has described oxalyl dicyanide. In 1972, the hydrolysis of diiminosuccinonitrile (DISN) was studied, and oxalyl dicyanide was obtained as an intermediate. The authors reported that colorless crystals of oxalyl dicyanide could be isolated upon controlled hydrolysis of DISN with two equivalents of *p*-toluenesulfonic acid monohydrate (*p*TsOH·H_2_O), followed by solvent removal and sublimation (see Scheme 1). A melting point of 61–62 °C was given, along with infrared, elemental, and mass spectrometric data. Oxalyl dicyanide was noted to be highly moisture sensitive, rapidly hydrolyzing to oxalic acid and hydrogen cyanide, and was therefore used in subsequent reactions without isolation. It was also reported to react with nucleophiles such as alcohols and amines, yielding the corresponding oxalate and oxamide derivatives by replacement of both nitrile groups.

**Scheme 1:**
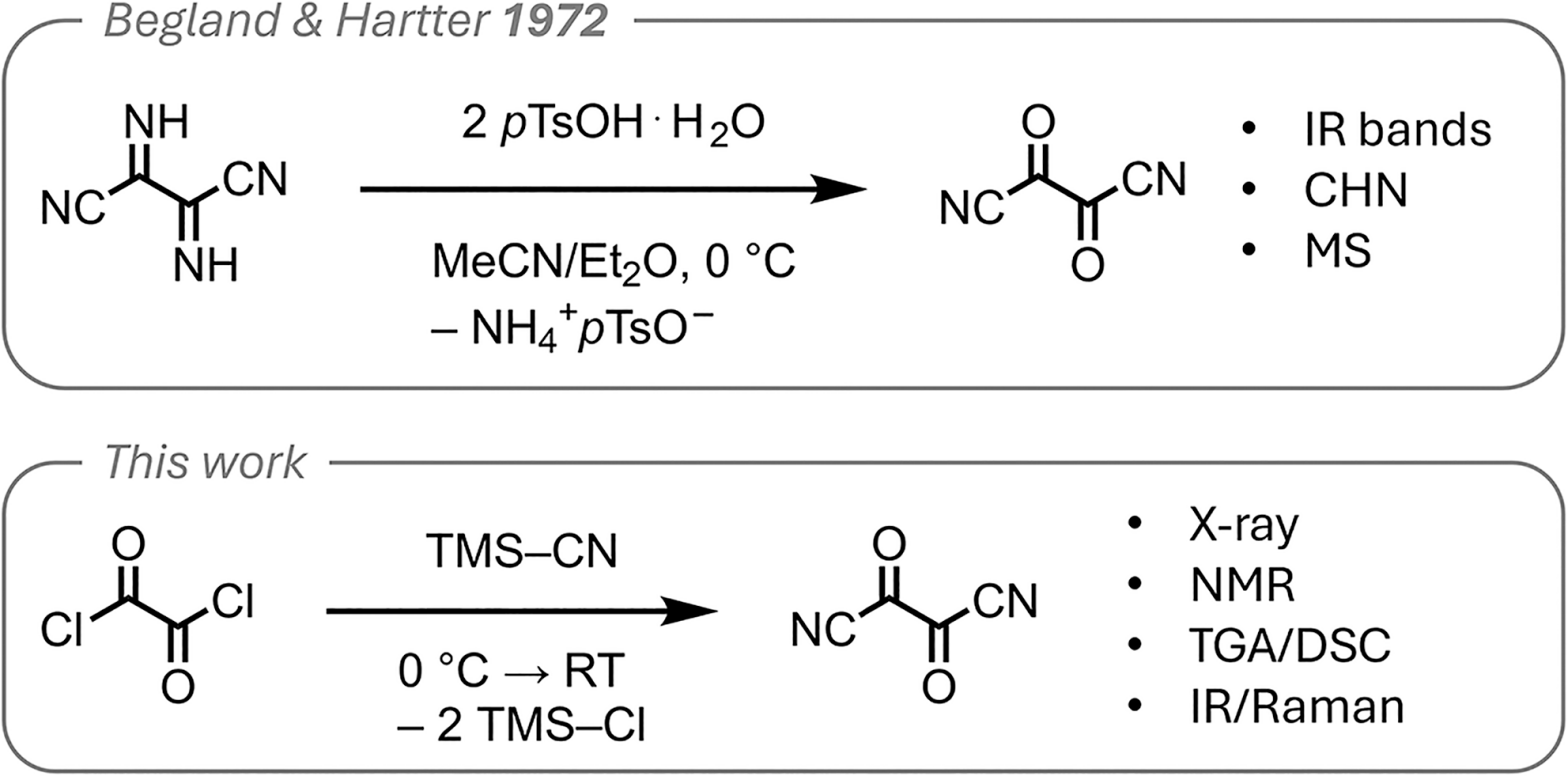
Top: Literature Known Synthesis of Oxalyl Dicyanide by Controlled Hydrolysis Starting from DISN and *p*TsOH·H_2_O; Bottom: Synthesis Used in This Work

In this work, we revisit the synthesis and characterization of oxalyl dicyanide by modern methodology. We report on the challenges associated with its synthesis and isolation, as well as the formation of its 1,4-dioxane addition product. Both compounds were characterized by single-crystal X-ray diffraction, NMR and vibrational spectroscopy, supported by quantum-chemical calculations. In addition, we determined the crystal structure of the hydrolysis product, the unusual tetraol 2,2,3,3-tetrahydroxysuccinonitrile, thereby expanding the chemistry of oxalyl pseudohalides and providing new insight into their structural diversity and reactivity.

## Results and Discussion

### Synthesis and Challenges

Our first attempts at synthesizing oxalyl dicyanide followed the procedure from literature.^4^ There, oxalyl dicyanide was obtained by controlled hydrolysis of diiminosuccinonitrile (DISN) with two equivalents of *p*TsOH·H_2_O. The synthesis was reported to be poor in yield if oxalyl dicyanide was isolated. Thus, it was used in subsequent reactions without isolation.

When we repeated the reaction, addition of *p*TsOH·H_2_O at 0 °C to DISN caused an immediate color change to yellow alongside the formation of a colorless precipitate. The resulting suspension turned orange and subsequently brownish/red within 10 min. After filtration, the solvent was removed under reduced pressure, leaving behind a sticky brown residue. The ^13^C NMR spectrum is given in the Supporting Information, Figure S10, indicating decomposition of the product. Immediate filtration after addition of *p*TsOH·H_2_O, while the suspension is still bright yellow, does not change the outcome. Attempts to sublimate oxalyl dicyanide from the brown residue yielded only a few crystals, which were unsuitable for single-crystal X-ray diffraction or further identification.

For the related carbonyl diisothiocyanate and thiocarbonyl dithiocyanate, metathesis reaction proved to be suitable.^7,9^ Thus, we applied this to the synthesis of oxalyl cyanide, starting from oxalyl chloride and AgCN. In contrast to the aforementioned syntheses, no reaction was observed in liquid sulfur dioxide at temperatures in the range of −30 to 0 °C. However, the reaction proceeded when the reaction was carried out in acetonitrile or tetrahydrofuran. In the latter case, immediately after addition of oxalyl chloride to a solution of AgCN in acetonitrile, a colorless precipitate of silver chloride formed, as verified by powder X-ray diffraction. Subsequently, the reaction mixture turned yellow and brownish after some time when the reaction was carried out at 0 °C to room temperature. Full conversion is achieved when oxalyl chloride is reacted with AgCN in acetonitrile at −40 °C for 6 h. Purification at low temperatures results in the precipitation of a slightly oily yellow to orange solid. While the ^13^C NMR spectrum of the reaction mixture shows two distinct signals at 160.4 and 105.8 ppm, the ^13^C NMR spectrum (see Supporting Information Figure S11) of the obtained product in THF-d8 and MeCN-d3 shows decomposition, multiple signals between 100 and 160 ppm and the formation of carbon monoxide at 185.2 ppm as the solution turns brown.

Since oxalyl chloride reacts with trimethylsilyl isothiocyanate to form oxalyl diisothiocyanate,[Fn fn1] we followed a similar procedure for our next attempts to synthesize oxalyl dicyanide, here starting from trimethylsilyl cyanide (TMS–CN). While oxalyl chloride does not react with TMS–CN in acetonitrile (confirmed by NMR), oxalyl chloride readily reacts with TMS–CN in toluene. After removal of all volatiles, this reaction yields a sticky orange oil. In the respective ^13^C NMR spectrum only one broad signal is present at 108.6 ppm. As it has been demonstrated that ZnI_2_ successfully catalyzes the preparation of aliphatic acyl cyanides from acid chloride and TMS–CN, we decided to try using ZnI_2_ as catalyst.^11^ By adding a catalytic amount of ZnI_2_ to the reaction mixture, one obtains a bright orange solution, which gives a colorless indefinable foam after filtration and removal of the volatile components (see Supporting Information, Figure S15).

Finally, oxalyl dicyanide can be obtained as a crystalline solid in the neat reaction of oxalyl chloride with TMS–CN at 0 °C in a 1:1 molar ratio, albeit in relatively poor yield. The reaction is given in Scheme 2. For the synthesis, oxalyl chloride is cooled to 0 °C and TMS–CN is added dropwise. Depending on the addition rate, the reaction mixture turns yellow within 2–10 min and a colorless crystalline solid forms at the surface. After full addition, the ice bath is removed and the reaction mixture stirred for another few minutes. The crude product can be purified in two steps: decanting the liquid and washing with *n*-hexane followed by drying in dynamic vacuum.

**Scheme 2:**
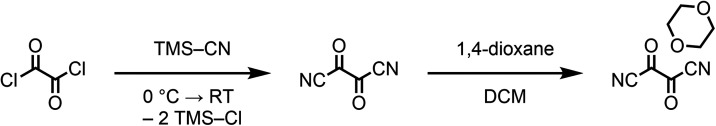
Synthesis of Oxalyl Dicyanide Starting from (COCl)_2_ and TMS–CN and the Formation of the 1,4-Dioxane Addition Product

It has been shown that oxalyl halides form addition compounds with 1,4-dioxane.^12,13^ Our attempts to increase the yield of the reaction by precipitating the oxalyl dicyanide adduct with 1,4-dioxane out of solution were unsuccessful. Nevertheless, when oxalyl dicyanide is dissolved in dichloromethane, the corresponding colorless dioxane adduct is formed as a solid upon addition of 1,4-dioxane at room temperature. The ^13^C NMR spectrum (DCM-d2, 300 K) of oxalyl dicyanide shows two signals at 159.8 ppm (C=O) and 109.9 ppm (CN). Similarly, the 1,4-dioxane addition product shows ^13^C NMR chemical shifts at 158.5 ppm (C=O) and 109.8 ppm (CN), respectively. The signal for the carbon atoms of 1,4-dioxane is located at 66.5 ppm.

Several attempts at using oxalyl dicyanide as starting material for subsequent chemical transformations were made. A literature precedent for carbonyl cyanide coordinating to transition metal ions exists.^14^ It was standing to reason that oxalyl dicyanide behaves similarly. However, all our attempts to form a stable and isolable adduct of oxalyl dicyanide with a range of metal ions (and a variety of counterions) proved unsuccessful; details can be found in the Supporting Information.

The only reference describing oxalyl dicyanide also contains a brief description of its follow-up chemistry.^4^ When generated in situ, the reaction of oxalyl dicyanide with nucleophiles proceeds under substitution of the CN moiety and forms HCN as side product. However, we were unsuccessful in repeating these experiments from the isolated oxalyl dicyanide and only obtained mixtures of reaction products that defied analysis. The search for a clean chemical transformation starting from oxalyl dicyanide is ongoing in our lab.

The thermal behavior of oxalyl dicyanide was studied with differential scanning calorimetry (DSC) and thermogravimetric analysis (TGA). The TGA-DSC data is shown in Figure 1, confirming the reported sublimation at elevated temperature. Oxalyl dicyanide shows thermal stability up to ∼80 °C. Thus, the DSC curve only shows one broad endothermic peak with an onset temperature of 80.9 °C (354.1 K) coinciding with an almost complete loss of mass in the TGA curve. The melting point of oxalyl dicyanide is reported at 61–62 °C along with a sublimation temperature of 50 °C at 0.5 mmHg.^4^

**Figure 1:**
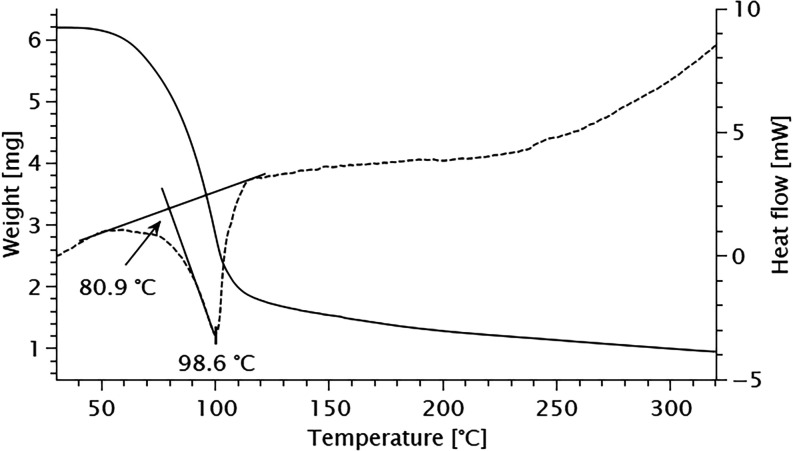
Thermogravimetric analysis (—) and differential scanning calorimetry (- - -) data of oxalyl dicyanide. For details of the fit see text.

The reaction is not selective and oxalyl dicyanide is only obtained as a side product in scarce yield of ∼20%. Moreover, the reaction cannot be appreciably scaled up. On a larger scale, the isolated yield does not increase significantly, while requiring the use of large amounts of toxic substances. Another major challenge is that oxalyl dicyanide is not obtained when two equivalents of TMS–CN are used in the reaction. Following the same procedure as described above, a color change to yellow is observed and a fluffy, almost colorless foam is obtained after removal of all volatile components. The ^13^C NMR spectrum does not show any signals corresponding to oxalyl dicyanide or any other species containing a carbonyl or cyanide moiety at all; specifically, no signals are observed above 5 ppm. Only a few small signals around 2.0 ppm suggest a TMS containing compound. The corresponding ^1^H NMR spectrum shows multiple broad overlapping signals between 0.33–0.67 ppm. The reaction product was further characterized by IR spectroscopy and TGA/DSC analysis (see Supporting Information Figure S4). In the TGA, only one thermal event can be observed at around 80 °C with almost complete loss of mass. In the DSC, one small exothermic process takes place at an onset temperature of 173 °C. The IR spectrum (see Supporting Information Figure S5) shows peaks at 2255, 1799, 1257–960 cm^–1^ and a very strong vibrational band at 832 cm^–1^. This information alone prevents unambiguous identification.

### Crystal and Molecular Structures

Single crystals of oxalyl dicyanide and its 1,4-dioxane addition product were picked directly from the reaction mixture. Oxalyl dicyanide crystallizes in the orthorhombic system with the centrosymmetric space group *Cmce* [No. 64 with *a* = 7.0770(9) Å, *b* = 7.5273(12) Å and *c* = 8.7580(10) Å at 100 K] and four formula units per unit cell (*Z* = 4). In the crystal structure, the molecule is disordered: Two molecules overlap, sharing the same O and N positions (see Figure 2). Consequently, both the O and N sites are fully occupied, whereas the two C atoms are only half occupied (constrained through space group symmetry). Thus, O and N are located on the special position with Wyckoff number 8*d* (2..), while C1 and C2 reside on the general Wyckoff position 16*g*. The oxalyl dicyanide molecule adopts *C*_2*h*_ symmetry with the carbonyl groups in *trans* orientation and the −CN moiety in *anti* position to the respective adjacent carbonyl groups. In the crystal lattice, individual molecules are arranged at each edge center and at the center of the unit cell, resulting in an AB stacking pattern along [001], in which the molecules are alternately tilted in an almost 45° degree angle. This arrangement results in a herringbone motif along [001] in which the carbonyl O atom of one molecule points toward the center of the C–C bond of a neighboring molecule in an adjacent layer (see Supporting Information Figure S29). Thus, the crystal packing is dominated by these strong dipole interactions with *d*_N···C_ = 2.816(2)/2.922(2) Å, as can be seen in the respective Hirshfeld surface (see Figure 2) and corresponding 2D fingerprint plots in the Supporting Information Figure S32. This N···C distance is shorter than the sum of their van der Waals radii (3.25) Å.^15^

**Figure 2:**
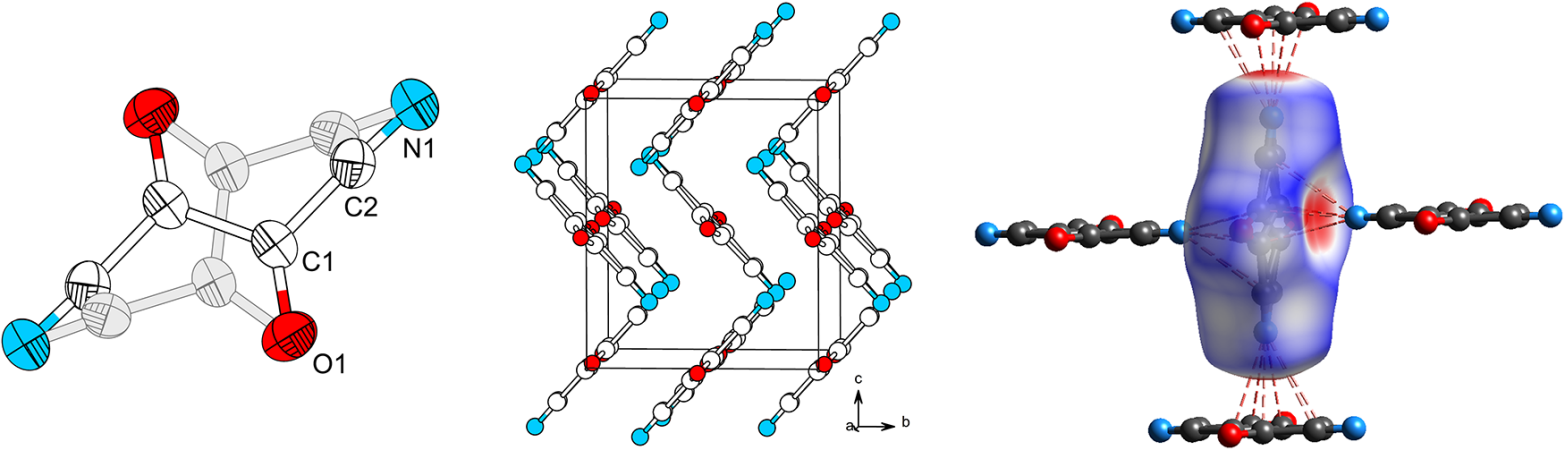
Left: Molecular structure of one disordered oxalyl dicyanide molecule in the solid state measured at 100 K. Atoms are drawn with 70% displacement ellipsoids. Middle: Unit cell of oxalyl dicyanide. Atoms are drawn with arbitrary radii. Right: Hirshfeld surface of oxalyl dicyanide at 100 K shown with neighboring molecules. Red areas indicate short contacts. Red dashed lines highlight N···C short contacts.

It has been shown that oxalyl halides form addition compounds with 1,4-dioxane.^12,13^ Attempts to increase the yield of oxalyl dicyanide by precipitating its corresponding 1,4-dioxane adduct yielded a colorless opaque precipitate of the mentioned compound, which contained crystals suitable for single crystal X-ray diffraction. The 1,4-dioxane addition product of oxalyl dicyanide crystallizes in the triclinic system with the space group *P*1̅ [No. 2 with *a* = 5.6902(6) Å, *b* = 6.8610(7) Å, *c* = 7.1350(7) Å, α = 111.665(7)°, β = 112.134(7)° and γ = 94.089(8)° at 100 K] and half a molecule in the asymmetric unit (*Z* = 1) (see Figure 3). All atoms occupy the Wyckoff position 2*i* (site symmetry 1). Similar to the solid formed between 1,4-dioxane and oxalyl fluoride,^13^ the crystal structure comprises infinite chains of alternating oxalyl dicyanide and 1,4-dioxane moieties along [111]. While the 1,4-dioxane is disordered at carbon positions C3A and C3B, the oxalyl dicyanide moiety is fully ordered, exhibiting a *trans* planar conformation with *C*_2*h*_ symmetry. The 1,4-dioxane shows the preferred chair conformation. In the chains, the distance from the 1,4-dioxane O atom to the nearest C-scaffold is 2.542(2)–3.169(2) Å, which is below the sum of their van der Waals radii (3.22 Å), indicating strong attractive intermolecular interactions.^15^ In addition, a weaker short contact between the distorted −CH_2_ moiety and the N atom of an oxalyl dioxane molecule in an adjacent chain can be observed.

**Figure 3:**
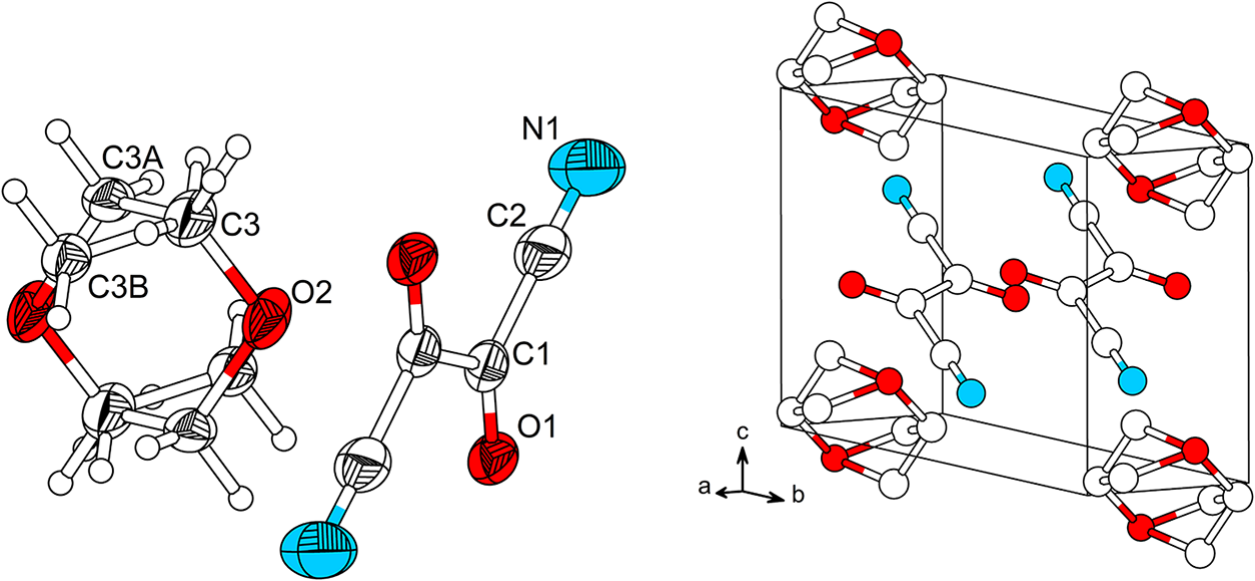
Left: Molecular structure of the 1,4-dioxane oxalyl dicyanide adduct in the solid state measured at 100 K. Atoms are drawn with 60% displacement ellipsoids. Right: Unit cell of 1,4-dioxane oxalyl dicyanide complex. Atoms are drawn with arbitrary radii. H-Atoms are omitted for clarity.

During our first attempts to determine the crystal structure of oxalyl dicyanide by single crystal X-ray diffraction, we indexed a unit cell different from the one described above. Structure solution and refinement showed, that two equivalents of water had been incorporated upon hydrolysis, yielding a unique tetraol, namely 2,2,3,3-tetrahydroxysuccinonitrile.

2,2,3,3-Tetrahydroxysuccinonitrile crystallizes in the monoclinic space group *P*2_1_/*n* [No. 14 with *a* = 7.1094(3) Å, *b* = 6.2431(2) Å, *c* = 7.3185(3) Å and β = 111.219(3)° at 100 K] with two formula units in the unit cell (*Z* = 2) and half a molecule in the asymmetric unit. The molecular structure is shown in Figure 4. In the crystal lattice, each corner and the center of the unit cell is occupied by one individual molecule with all atoms residing on the general Wyckoff position 4*e* (site symmetry 1). Both central C atoms of the former oxalyl dicyanide now bear two hydroxy groups and a cyanide moiety simultaneously, representing an unusual exception to the Erlenmeyer rule. This rule states that organic compounds carrying more than one hydroxy group on the same C atom are inherently unstable and tend to dehydration.^16^ Prominent exceptions are those with strong electron withdrawing groups in the α-position, such as ninhydrin (2,2-dihydroxyindane-1,3-dione) or chloral hydrate.^17,18^ Attempts at a targeted synthesis of the compound proved unsuccessful. Both slow hydrolysis of oxalyl dicyanide upon exposure to air and controlled hydrolysis with stoichiometric amounts of water in dichloromethane or chloroform resulted in decomposition into uncharacterizable products. Consequently, systematic preparation of the tetraol could not be achieved. Experimental details for the procedures can be found in the Supporting Information.

**Figure 4:**
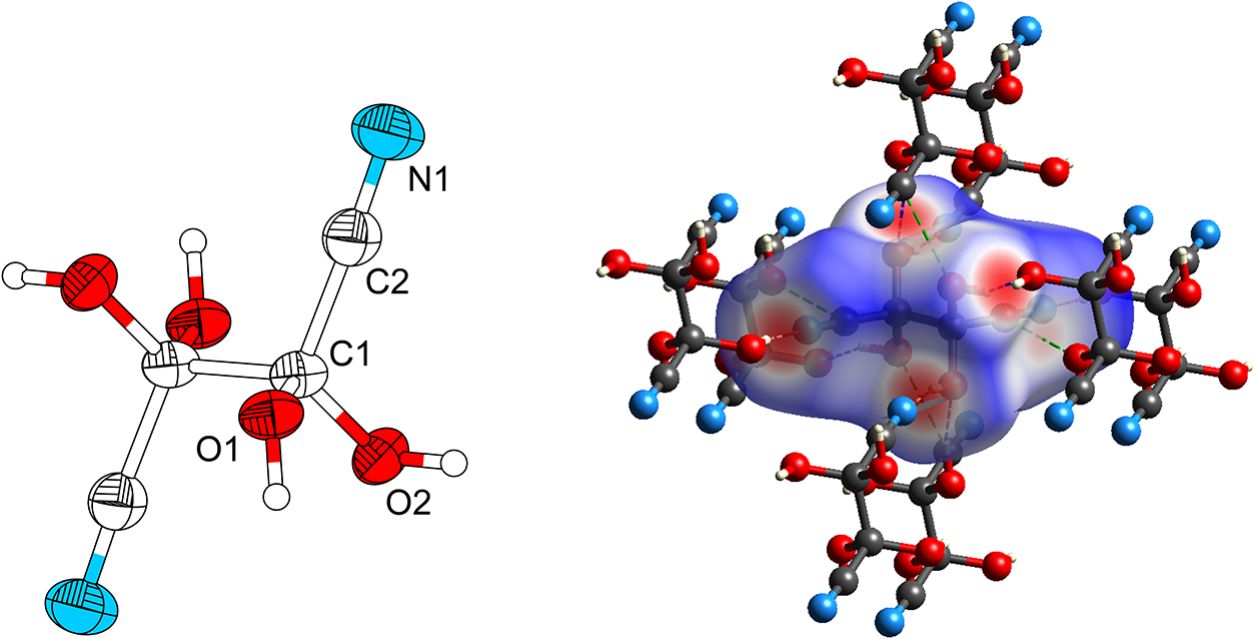
Left: Molecular structure of one 2,2,3,3-tetrahydroxysuccinonitrile molecule in the solid state measured at 100 K. Atoms are drawn with 80% displacement ellipsoids. Right: Hirshfeld surface of 2,2,3,3-tetrahydroxysuccinonitrile at 100 K shown with neighboring molecules. Red areas indicate short contacts. Blue dashed lines highlight O···H short contacts and green dashed lines highlight O···C short contacts.

As shown by Hirshfeld surface analysis (Figure 4 right), the crystal packing is dominated by strong hydrogen bonding between the hydroxy group and either an adjacent hydroxy or cyanide group. The intermolecular distances are *d*_OH···O_ = 1.95(2) Å and *d*_OH···N_ = 1.95(3) Å and thus below their corresponding van der Waals radii 2.622 Å and 2.265 Å, respectively. Weaker C···O dipole interactions are also present, as can be seen in the Hirshfeld surface.

Selected structural parameters of oxalyl dicyanide, its 1,4-dioxane addition product, and 2,2,3,3-tetrahydroxysuccinonitrile are summarized in Table 1. The bond lengths in the oxalyl cyanide molecules are very similar for pure oxalyl dicyanide and its dioxane addition product. Only the C=O bond length of 1.225(3) Å in oxalyl dicyanide is slightly longer than in the corresponding 1,4-dioxane complex (1.196(2) Å). The same observation was made for oxalyl chloride and bromide in which the C=O distance decreased by 0.143 and 0.055 Å, respectively, when cocrystallized with 1,4-dioxane.^12^ The −CN moiety is essentially linear, with angles of 177.3(5)° and 178.9(2)° for oxalyl dicyanide and its dioxane adduct, respectively. The corresponding C1–C2 and C2≡N1 distances amount to 1.488(3)/1.486(2) Å and 1.130(3)/1.126(2) Å. Comparable geometric parameters have also been observed in carbonyl dicyanide cocrystallized with AgSbF_6_.^14^ In this case, the ∠(C–C≡N) is 178.5(3)°, with bond distances of *d*(C–C) = 1.469(4) Å and *d*(C≡N) = 1.139(2) Å, respectively.

**Table 1:** Comparison of Selected Interatomic Distances of Oxalyl Dicyanide, Its 1,4-Dioxane Addition Complex and 2,2,3,3-Tetrahydroxysuccinonitrile with Chosen Compounds for Comparison^a^

	oxalyl dicyanide	1,4-dioxane complex	2,2,3,3-tetrahydroxysuccinonitrile	carbonyl dicyanide^14^
C=O	1.225(3)	1.196(2)	1.3754(14)/1.3919(14)	1.184(4)
C1–C1	1.523(4)	1.524(3)	1.561(2)	–
C1–C2	1.488(3)	1.486(2)	1.507(2)	1.469(4)^b^
C≡N	1.130(3)	1.126(2)	1.139(2)	1.130(4)^b^
∠C–C≡N	177.3(5)	178.9(2)	176.22(13)	178.5(3)^b^

a Parameters in [Å] and [°].

b Mean value.

As seen from Table 1, the bond lengths in 2,2,3,3-tetrahydroxysuccinonitrile are noticeably longer than those in **1** and **2**. The C1–C1 and C1–C2 distances amount to 1.561(2) and 1.507(2) Å, respectively, and are thus slightly elongated compared to textbook values for sp^3^–sp^3^ (1.53 Å) and sp^3^–sp (1.47 Å) single bonds.^19^ In contrast, the C–O bond lengths of 1.3754(14)/1.3919(14) Å are shorter than a typical single bond (1.43 Å), yet fall within the range reported for other diols, such as 1.358(5)/1.414(4) Å in ninhydrin and 1.384(5)/1.407(5) Å in alloxan tetrahydrate.^17,20^ Furthermore, the ∠(C–C≡N) angle is slightly more bent at 176.22(13)°, and the C≡N bond lengths are marginally longer at 1.139(2) Å compared to those in oxalyl dicyanide.

### Vibrational Spectroscopy

The room temperature Raman and IR spectra are shown in Figure 5 and compared to its calculated spectra at the DFT-PBE0-D3/TZVP level of theory. For the calculations, a fully ordered model is required, resulting in space group *P*2_1_2_1_2_1_ (No. 19) after two *klassengleiche* symmetry reductions of index 2 from *Cmce* (No. 64) and *Pbca* (No. 61). The Bärnighausen tree is shown in the Supporting Information in Figure S38.

**Figure 5:**
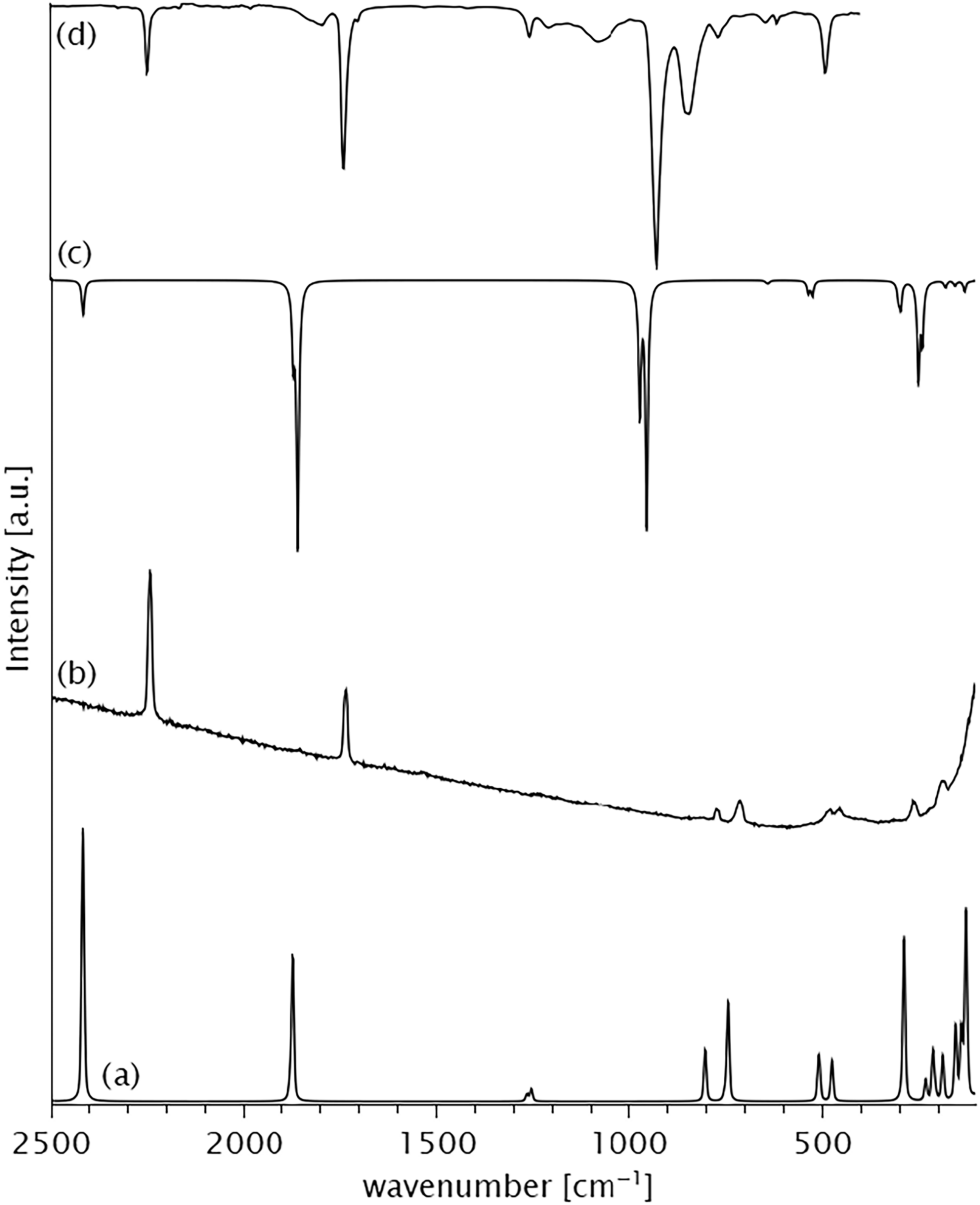
Calculated and experimental Raman (a and b) and IR spectra (c and d) of oxalyl dicyanide at room temperature.

The calculated and experimental IR spectra show excellent agreement, lending strong support to the molecular geometry established by X-ray diffraction. Table S5 in the Supporting Information lists the vibrational frequencies together with the assignment of each normal mode. For additional details, see the experimental section. Overall, both spectra show only a few distinct vibration bands, but the IR spectrum also shows vibration bands of a second species. The low energy region is dominated by the characteristic C≡N and C=O stretching vibration observed at 2243 and 1734 cm^–1^ in the Raman spectrum and at 2248 and 1735 cm^–1^ in the corresponding IR spectrum. At ∼1260 cm^–1^ in the calculated Raman spectrum the symmetric C–C stretch is located, too weak to be observed in the experimental spectrum and also IR inactive. In the IR spectrum, only two more modes can be observed below 1000 cm^–1^. First, at 923 cm^–1^ the antisymmetric C–CN stretch together with scissoring of the whole scaffold is found. Second at 487 cm^–1^, out-of-plane wagging of the carbon framework is observed. In the Raman spectrum below 1000 cm^–1^ several bands attributed to deformation vibrations are observed. At 774 cm^–1^ twisting of the C–C bond; at 712 cm^–1^ bending of the whole carbon framework and at 261 cm^–1^ the bending of the C≡N groups are found.

The assignment of the vibrational spectra for the corresponding 1,4-dioxane adduct is similar to the parent dicyanide. Characteristic vibrational bands are observed at ν­(C=O) (2238 cm^–1^), ν­(C≡N) (1728 cm^–1^), ν_as_(C–CN) (929 cm^–1^) and δ­(N≡C–C=O) (501 cm^–1^) for the oxalyl dicyanide fragment in the IR spectrum (see Supporting Information). Extra features for the 1,4-dioxane counterpart are in agreement with literature data.^21^

## Conclusion

Oxalyl dicyanide has been accessed as a crystalline solid from the reaction of oxalyl chloride with trimethylsilyl cyanide, and its propensity to form a stable 1,4-dioxane adduct has been demonstrated. The compound is obtained only in modest yield and presents significant challenges in synthesis, isolation, and handling. Hydrolytic instability leads to oxalic acid and hydrogen cyanide, with the transient formation of 2,2,3,3-tetrahydroxysuccinonitrile, a rare tetraol that constitutes an exception to the Erlenmeyer rule. Structural analysis reveals *C*_2*h*_ symmetry in the solid state, with nitrile groups oriented *anti* to the carbonyl moieties, while complementary spectroscopic and quantum chemical data provide consistent support for the crystallographic assignment. Together, these findings expand the chemistry of oxalyl derivatives and highlight both the opportunities and limitations in accessing highly reactive cyanide-containing species.

## Experimental Section

### General Remarks

All reactions and manipulations were carried out under an inert argon atmosphere using standard Schlenk-line or glovebox techniques (MBraun UNIlab glovebox, maintained at <0.5 ppm of H_2_O and <0.5 ppm of O_2_). Oxalyl chloride (Fisher Scientific GmbH, 98%) and trimethylsilyl cyanide (Fisher Scientific GmbH, 98%) were recondensed and degassed before use. 1,4-Dioxane was taken from a storage bottle and its purity checked by NMR. Solvents were dried following established procedures.^22^ DCM-d2 (Eurisotop, 99.5%) and CDCl_3_ (Eurisotop, 99.5%) were recondensed, degassed and stored over molecular sieve (4 Å) prior to use.

### Synthesis

#### Oxalyl Dicyanide

In a neat reaction, oxalyl chloride (0.43 mL, 5.00 mmol, 2.00 equiv) was cooled to 0 °C and trimethylsilyl cyanide (0.31 mL, 2.5 mmol, 1.00 equiv) was added dropwise. After full addition, the ice bath was removed, and the suspension was stirred for another 20 min. A color change from colorless to yellow was observed within 2–10 min along with the formation of a colorless precipitate. The solution was decanted and the residue washed with *n*-hexane (2 ·2 mL). Oxalyl dicyanide (56.0 mg, 518 μmol) was obtained as a colorless to yellow crystalline solid in 21% yield. ^13^C NMR (75 MHz, DCM-d2, 300 K): δ/ppm = 159.7 (C=O), 109.9 (−CN).

#### Oxalyl Dicyanide 1,4-Dioxane Adduct

Oxalyl dicyanide was prepared in situ as described above, starting from oxalyl chloride (0.43 mL, 5.00 mmol, 2.00 equiv) and TMS–CN (0.31 mL, 2.50 mmol, 1.00 equiv). The oily crude product was dissolved in 1.00 mL dichloromethane and subsequently excess 1,4-dioxane (0.21 mL, 2.50 mmol, 1.00 equiv) was added at room temperature. Immediately, the formation of a colorless solid was observed. After 10 min, all volatile components were removed under dynamic vacuum and the oily crude product was washed with *n*-hexane (1 mL). The oxalyl dicyanide 1,4-dioxane addition product was obtained as a colorless solid (167 mg, 0.85 mmol, 68%). ^13^C NMR (75 MHz, DCM-d2, 300 K): δ/ppm = 158.5 (C=O), 109.8 (−CN).

### NMR Spectroscopy

^13^C NMR spectra were acquired on a Bruker Avance II (300 MHz) or Avance III HD (300 MHz) spectrometer at 300 K if not stated otherwise.

### Infrared Spectroscopy

IR spectra were recorded on a Bruker Alpha FT-IR spectrometer equipped with a diamond ATR unit mounted in a nitrogen-filled glovebox (MBraun UNIlab glovebox, maintained at <0.1 ppm of H_2_O and <0.1 ppm of O_2_).

### Raman Spectroscopy

Raman spectra were recorded on a Monovista CRS+ confocal Raman microscope (Spectroscopy & Imaging GmbH) using a 532 nm solid-state laser and a 300 grooves/mm grating if not stated otherwise. Samples were flame-sealed in borosilicate capillaries and measured at room or low temperature.

### Hirshfeld Surface Analyses

Hirschfeld surface analyses were conducted with the Crystal Explorer (version 21.5) software suite.^23,24^

### TGA/DSC Measurements

High temperature TGA and DSC measurements were performed by the in-house service personnel using a DSC-TGA 3 (Mettler Toledo) device in a nitrogen-filled glovebox.

### Single Crystal X-ray Structure Determination

Single-crystal X-ray diffraction data were collected using a StadiVari (Stoe, Darmstadt) diffraction system equipped with mirror monochromated Cu Kα radiation (λ = 1.54186 Å, Xenocs Microfocus Source) and a Pilatus 300 K detector. Crystals were selected under Paratone-N oil, mounted on micromount loops and quench-cooled using an Oxford Cryosystems open flow N2 cooling device. If not stated otherwise, data were collected at 100 K and processed using the X-Area program package, including unit cell parameter refinement and interframe scaling (which was carried out using LANA within X-Area). Structures were subsequently solved using direct methods (SHELXT)^25^ and refined on *F*2 with SHELXL^26^ using the Olex2 ^27^ user interface. The crystal structure drawings were generated with DIAMOND.^28^

### Calculations with Periodic Boundary Conditions

Periodic quantum-chemical calculations were carried out for all discussed compounds with the PBE0 hybrid density functional theory method (DFT-PBE0).^29,30^ All calculations were performed with the CRYSTAL17 program package.^31^ Triple-ζ-valence + polarization (TZVP) level basis sets were applied for H, C, N and O. The basis sets have been previously derived from the Karlsruhe def2 basis sets.^32^ Intermolecular van der Waals dispersion interactions were taken into account using Grimme’s empirical D3 dispersion correction with zero damping.^33^ Reciprocal space was sampled by Monkhorst–Pack type k-meshes for oxalyl dicyanide: 4 × 4 × 3, 1,4-dioxane oxalyl dicyanide: 5 × 4 × 4 and 2,2,3,3-tetrahydroxysuccinonitrile: 4 × 5 × 4.^34^ For the evaluation of the Coulomb and exchange integrals (TOLINTEG), tolerance factors of 8, 8, 8, 8, and 16 were used for all calculations. Atomic positions and lattice parameters were fully optimized considering the space group symmetry. Default DFT integration grids and optimization convergence thresholds were applied in all calculations. The harmonic vibrational frequencies and Raman and IR intensities were obtained using CRYSTAL.^35^ Raman and IR intensities were calculated for a polycrystalline powder sample (total isotropic intensity in arbitrary units). The Raman spectra were obtained by using a pseudo-Voigt band profile (50:50 Lorentzian:Gaussian) and a full width at half-maximum (FWHM) of 8 cm^–1^ and simulated taking into account the experimental setup (293.15 K; λ = 532 nm). Bands for the Raman spectrum were assigned by visual inspection of the normal modes with the program package at http://crysplot.crystalsolutions.eu/.^36^

## Supplementary Material



## Footnotes

1 Our own observations, unpublished.

## Notes

Additional crystal structure and reaction pictures, Hirshfeld surface analysis, infrared and Raman data, quantum chemical data (PDF)
