# Supplementary material for: Preparation and Characterization of Oxalyl Dicyanide
Source: Inorg Chem. 2026 Feb 20;65(30):17334–41. doi: 10.1021/acs.inorgchem.5c05620 (PMC13439657; doi:10.1021/acs.inorgchem.5c05620)
Supplement: Supplementary file 1 [file ic5c05620_si_001.pdf]

Supporting Information

On the preparation and characterization of oxalyl dicyanide

Sven Ringelband[1], Theodore Cohen[1] and Frank Tambornino*[1]

Department of Chemistry, Philipps-University Marburg, Marburg

(E-mail: frank.tambornino@chemie.uni-marburg.de)

Contents

1. Additional analytical data 2
   - 1.1. Oxalyl dicyanide 2
   - 1.2. Side product 3
   - 1.3. 1,4-Dioxane oxalyl dicyanide complex 4
   - 1.4. Others 6
2. Additional pictures 8
3. Reactivity studies 10
4. Crystallographic data 12
   - 4.1. Additional Pictures for oxalyl dicyanide 13
   - 4.2. Additional Pictures for the 1,4-dioxane oxalyl dicyanide complex 14
   - 4.3. Additional Pictures for 2,2,3,3-tetrahydroxysuccinonitrile 15
5. Additional details on Hirshfeld surface analysis 17
   - 5.1. Oxalyl dicyanide 17
   - 5.2. 1,4-Dioxane oxalyl dicyanide complex 18
   - 5.3. 2,2,3,3-Tetrahydroxysuccinonitrile 19
6. Details on quantum chemical calculations 21

1. Additional analytical data

1.1. Oxalyl dicyanide

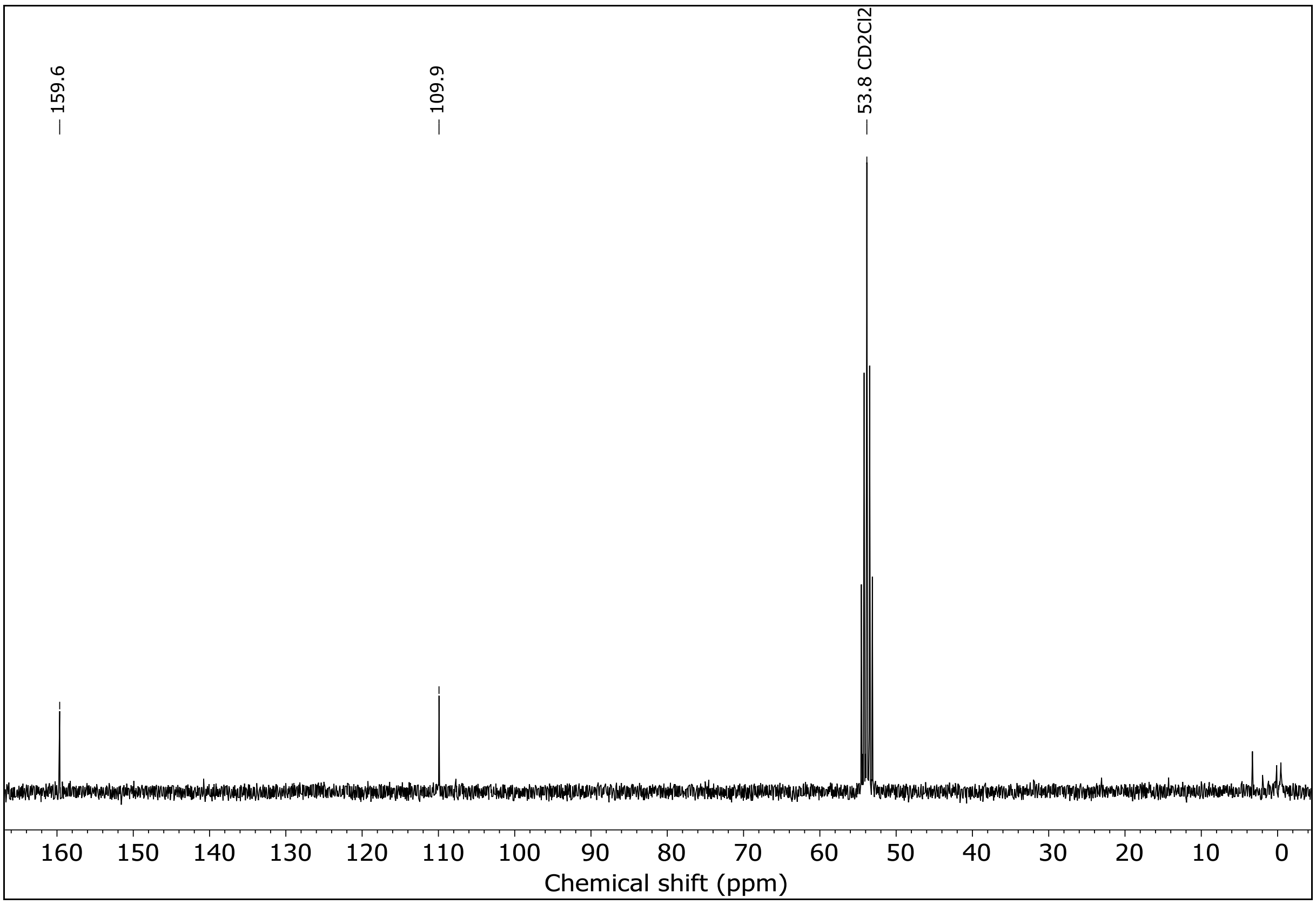

Figure S 1: ^{13}C NMR spectrum of oxalyl dicyanide in DCM-d2 at room temperature.

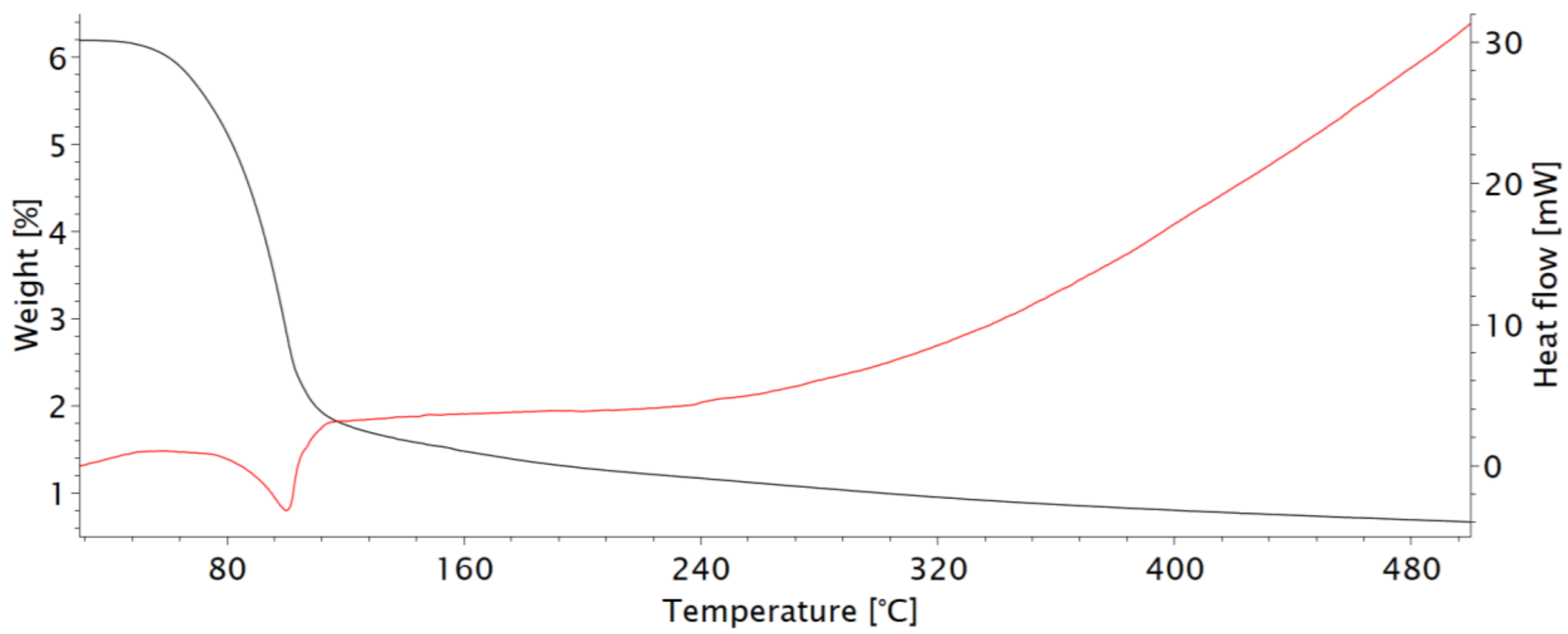

Figure S 2: Differential scanning calorimetry (red) and thermogravimetric analysis (black) of oxalyl dicyanide. Heating rate: 5 K min^{-1}. (T_{onset} = 80.9 °C and T_{peak} = 98.6 °C).

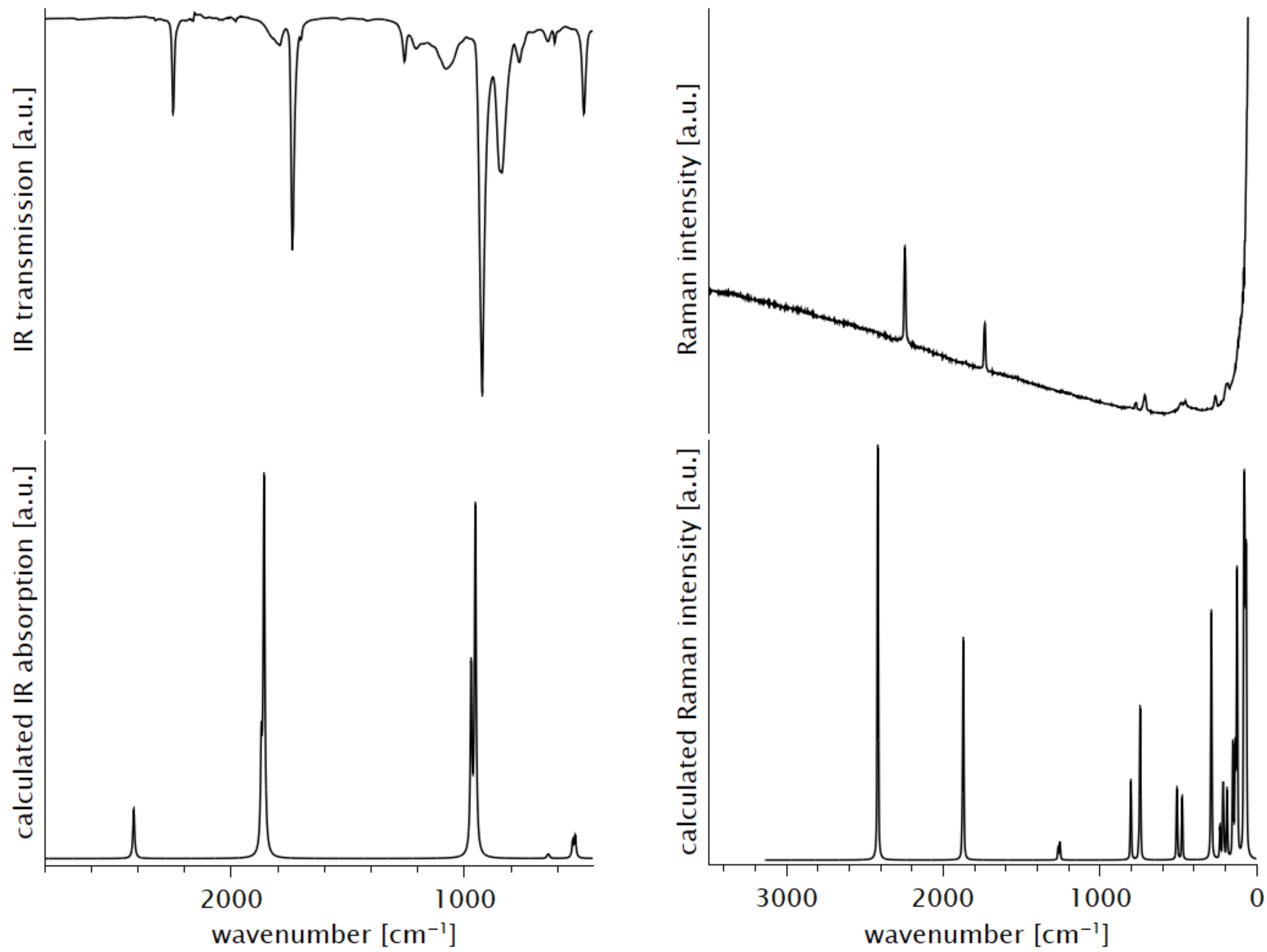

Figure S 3: Left: Measured (top) and calculated (bottom) solid-state room-temperature ATR-IR spectrum of oxalyl dicyanide Right: Measured (top) and calculated (bottom) solid-state room-temperature Raman spectrum of oxalyl dicyanide. Band assignments can be found in Table S 6 and
Table S **5**.

1.2. Side product

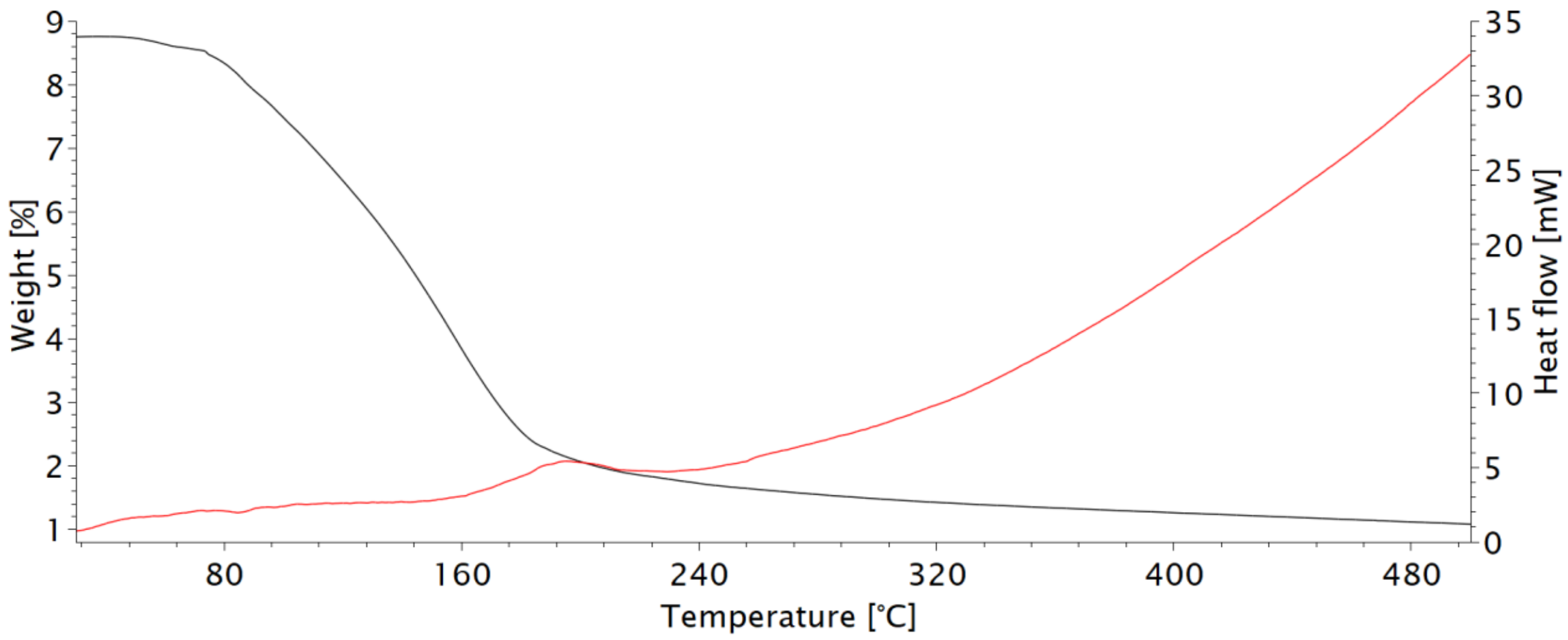

Figure S 4: Differential scanning calorimetry (red) and thermogravimetric analysis (black) of the reaction product of oxalyl chloride and TMS–CN in a 1 : 2 ratio. Heating rate: 5 K min^{-1}.

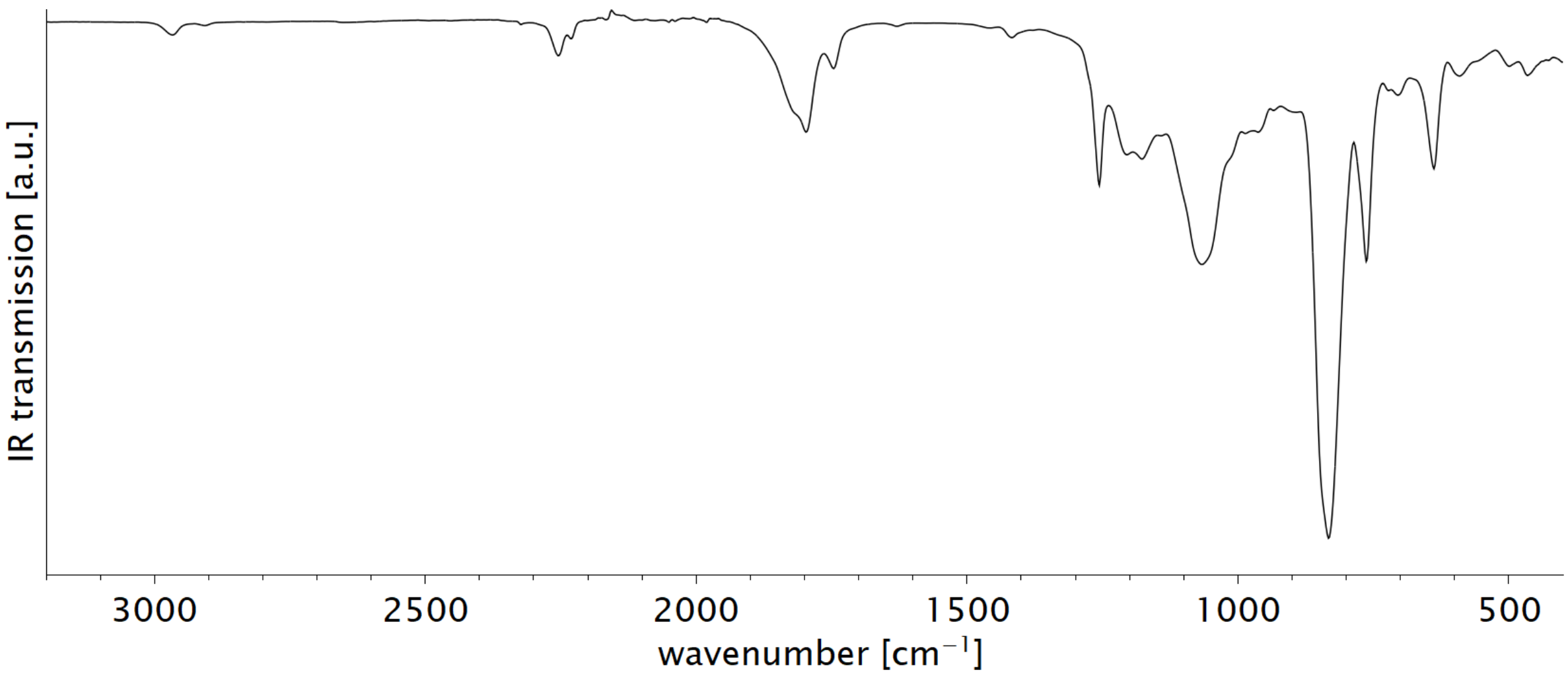

Figure S 5: Room temperature IR spectrum of the reaction product of oxalyl chloride and TMS–CN in a 1 : 2 ratio.

1.3. 1,4-Dioxane oxalyl dicyanide complex

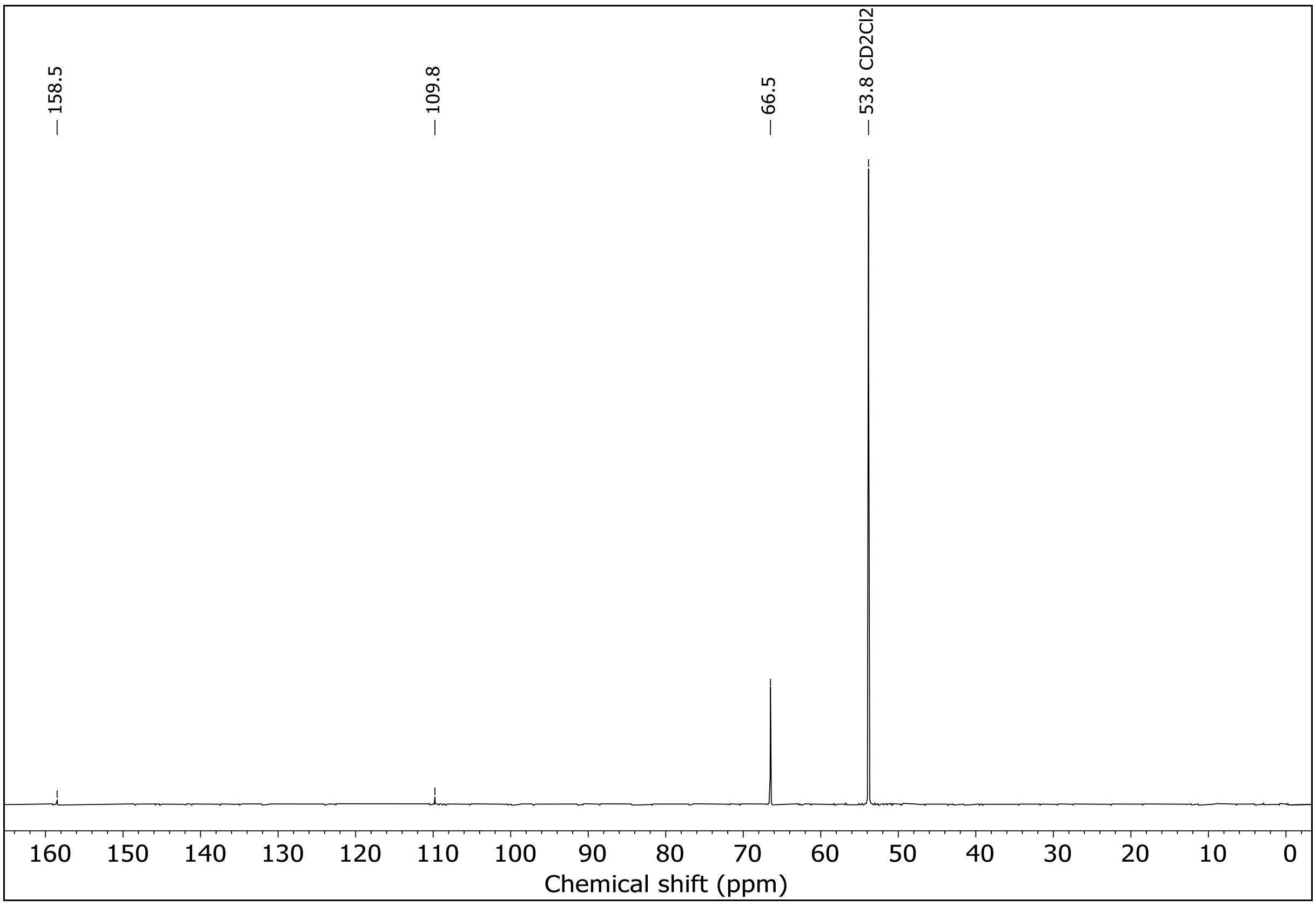

Figure S 6: ^{13}C NMR spectrum of the oxalyl dicyanide 1,4-dioxane complex in DCM-d2 at room temperature.

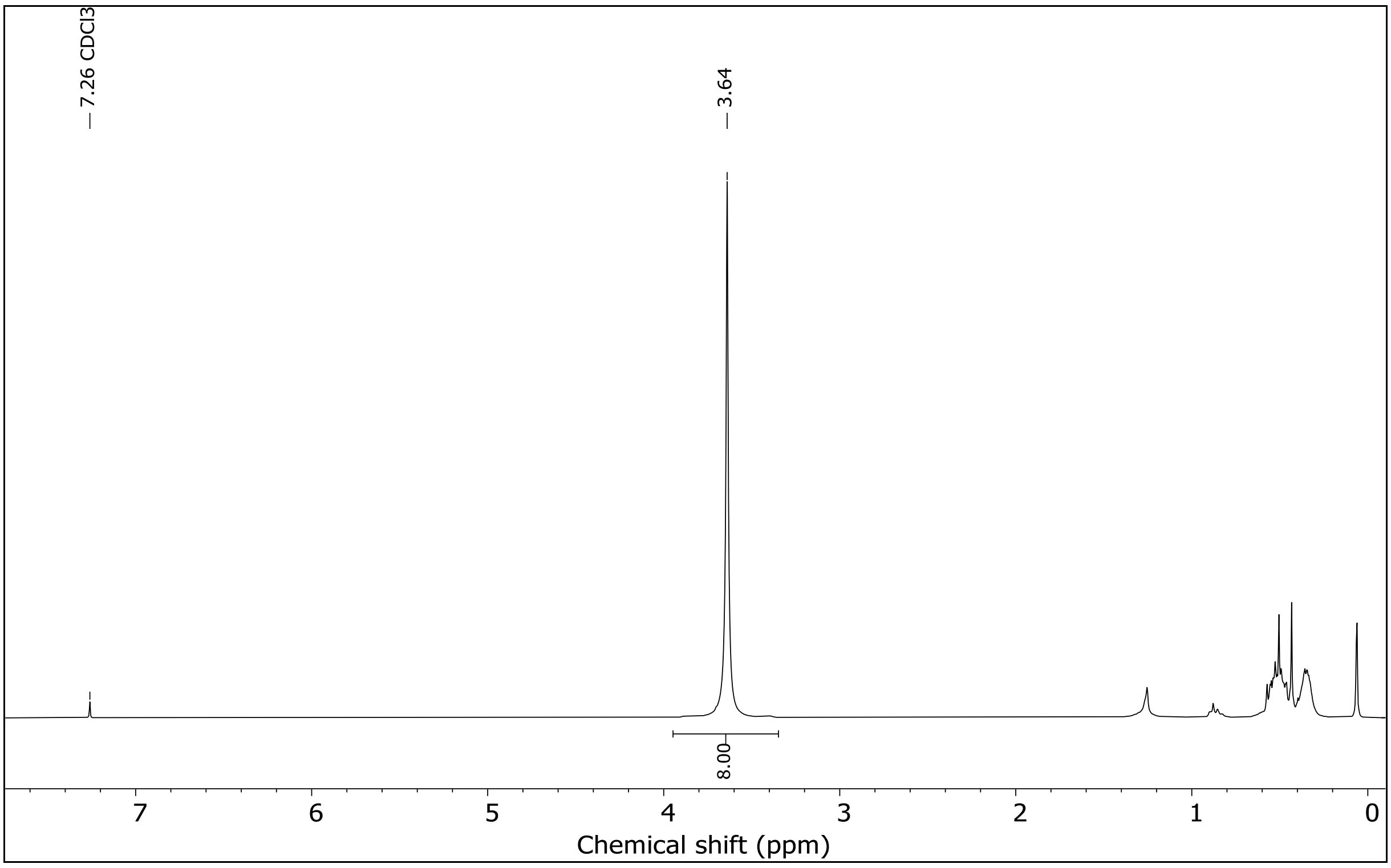

Figure S 7: ^{1}H NMR spectrum of the oxalyl dicyanide 1,4-dioxane adduct in $CDCl_3$ at room temperature. Minor impurity of TMS species.

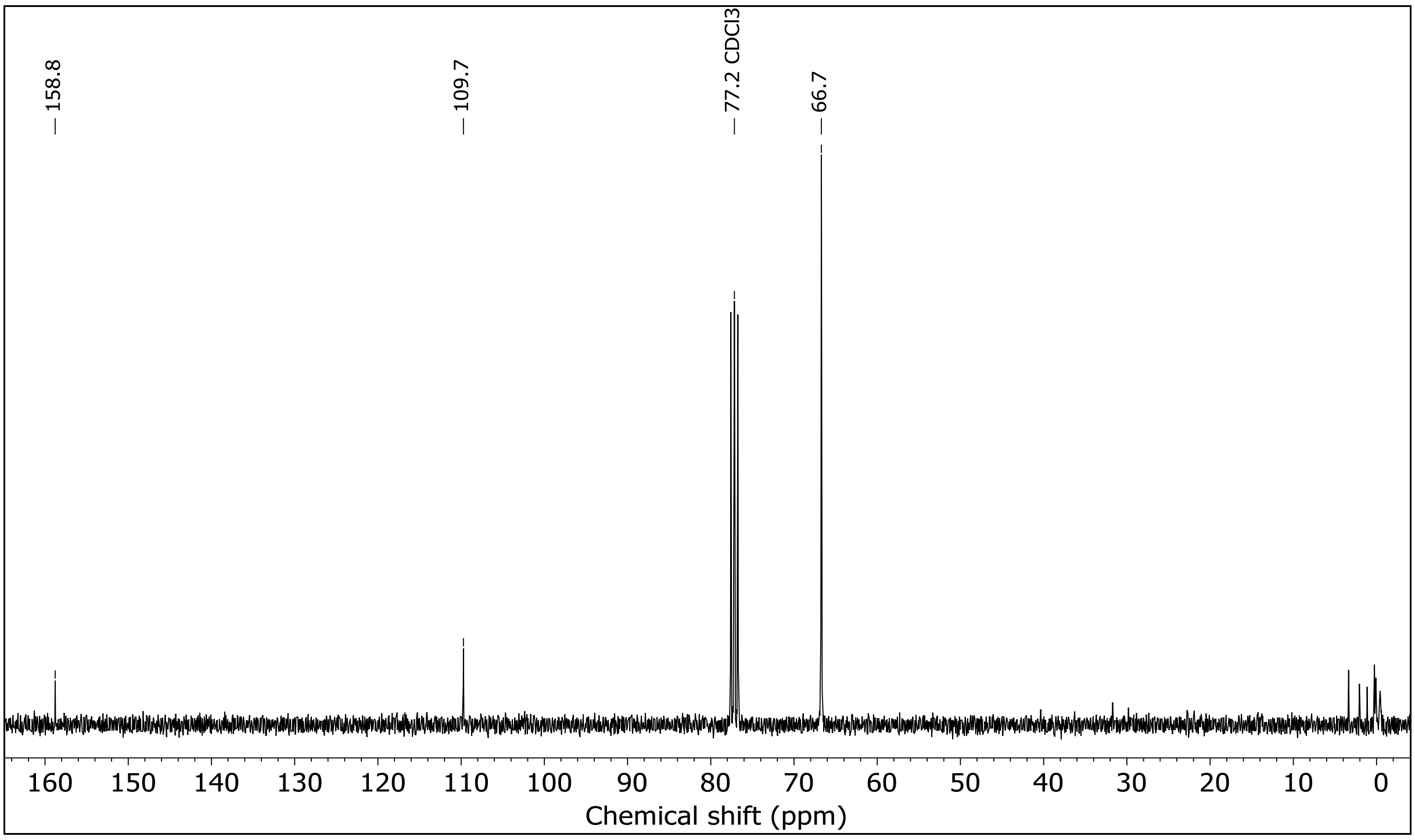

Figure S 8: ^{13}C NMR spectrum of the oxalyl dicyanide 1,4-dioxane adduct in $CDCl_3$ at room temperature. Minor impurities of TMS species.

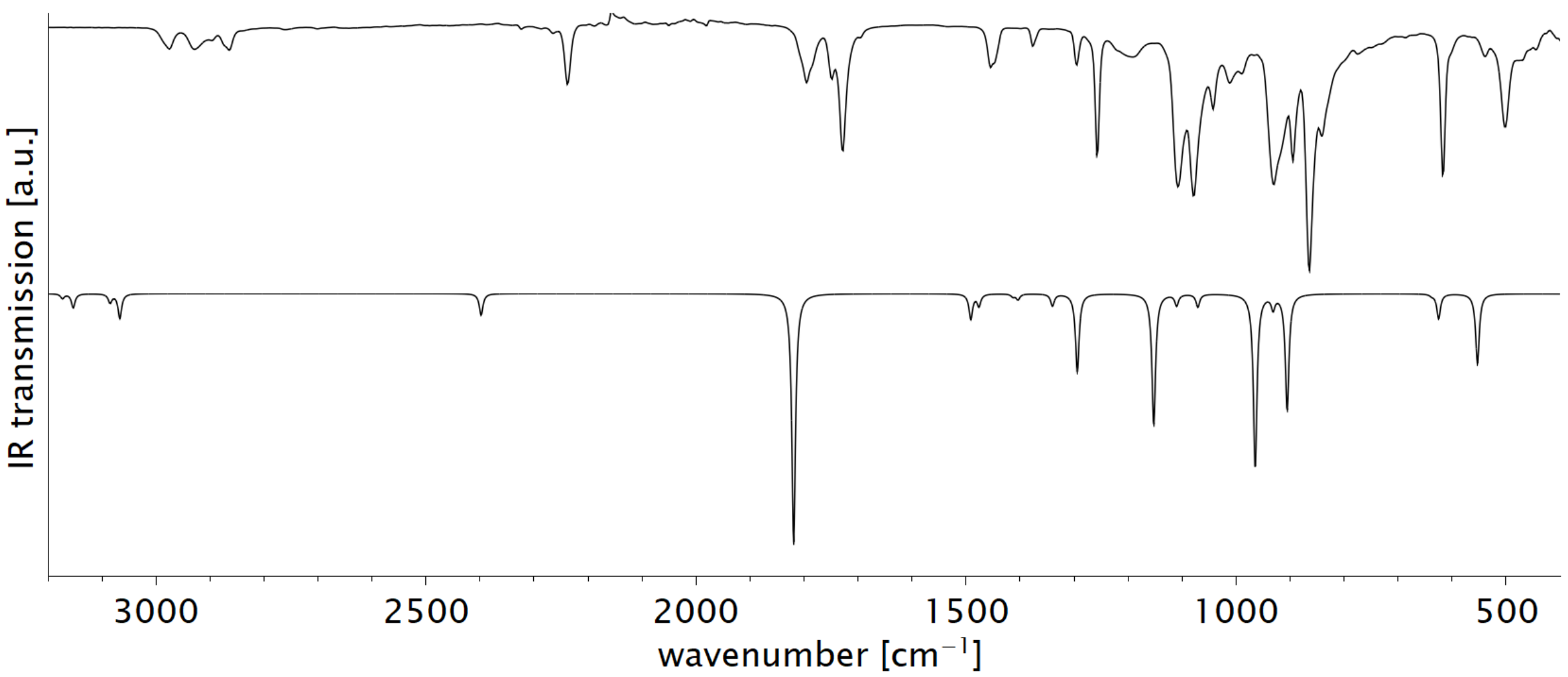

Figure S 9: Measured (top) and calculated (bottom) solid-state room temperature ATR-IR spectrum of the oxalyl dicyanide 1,4-dioxane adduct.

1.4. Others

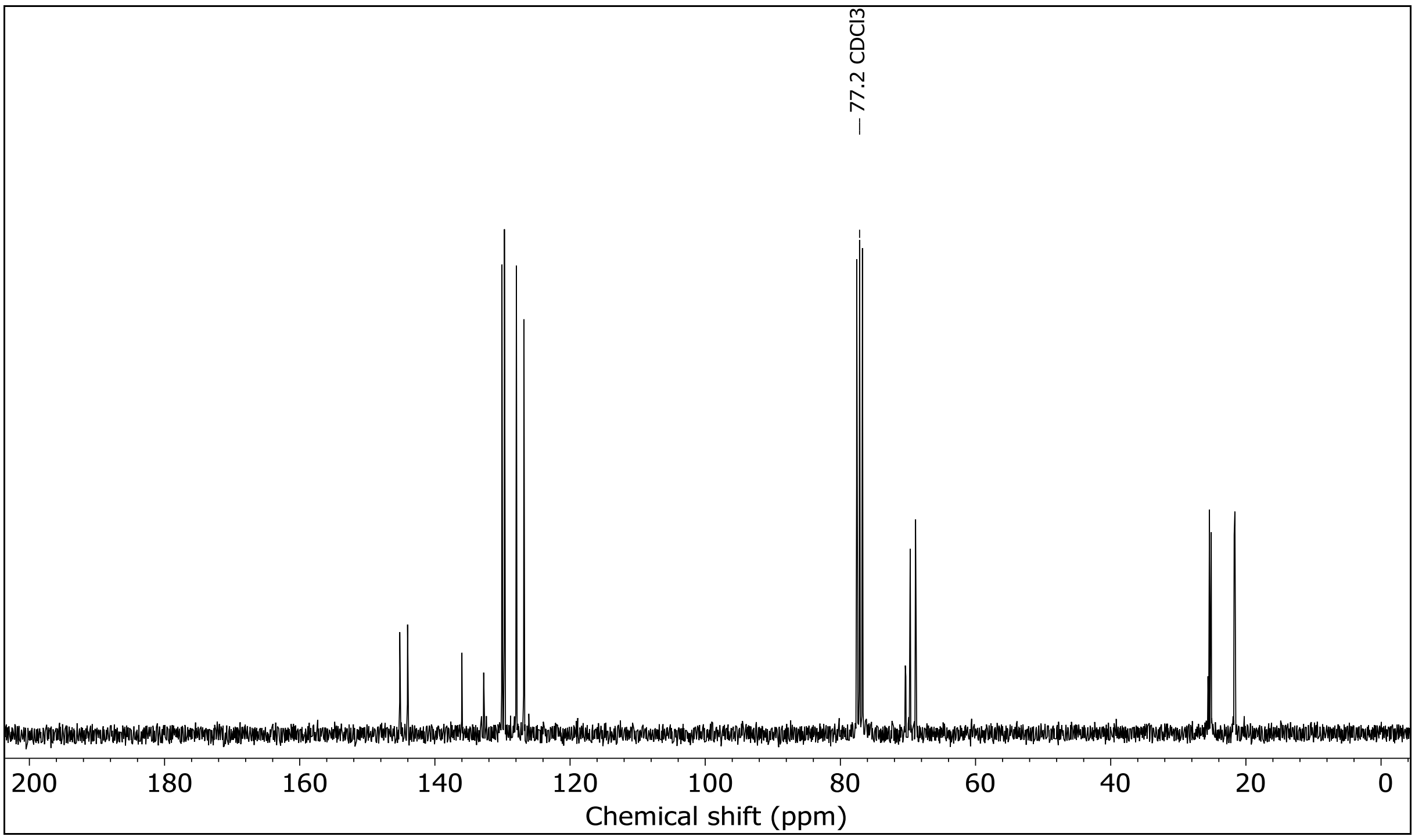

Figure S 10: ^{13}C NMR spectrum in $CDCl_3$ of the reaction product of *p*TsOH·H_2O and DISN at 0 °C after filtration and removal of the solvent.

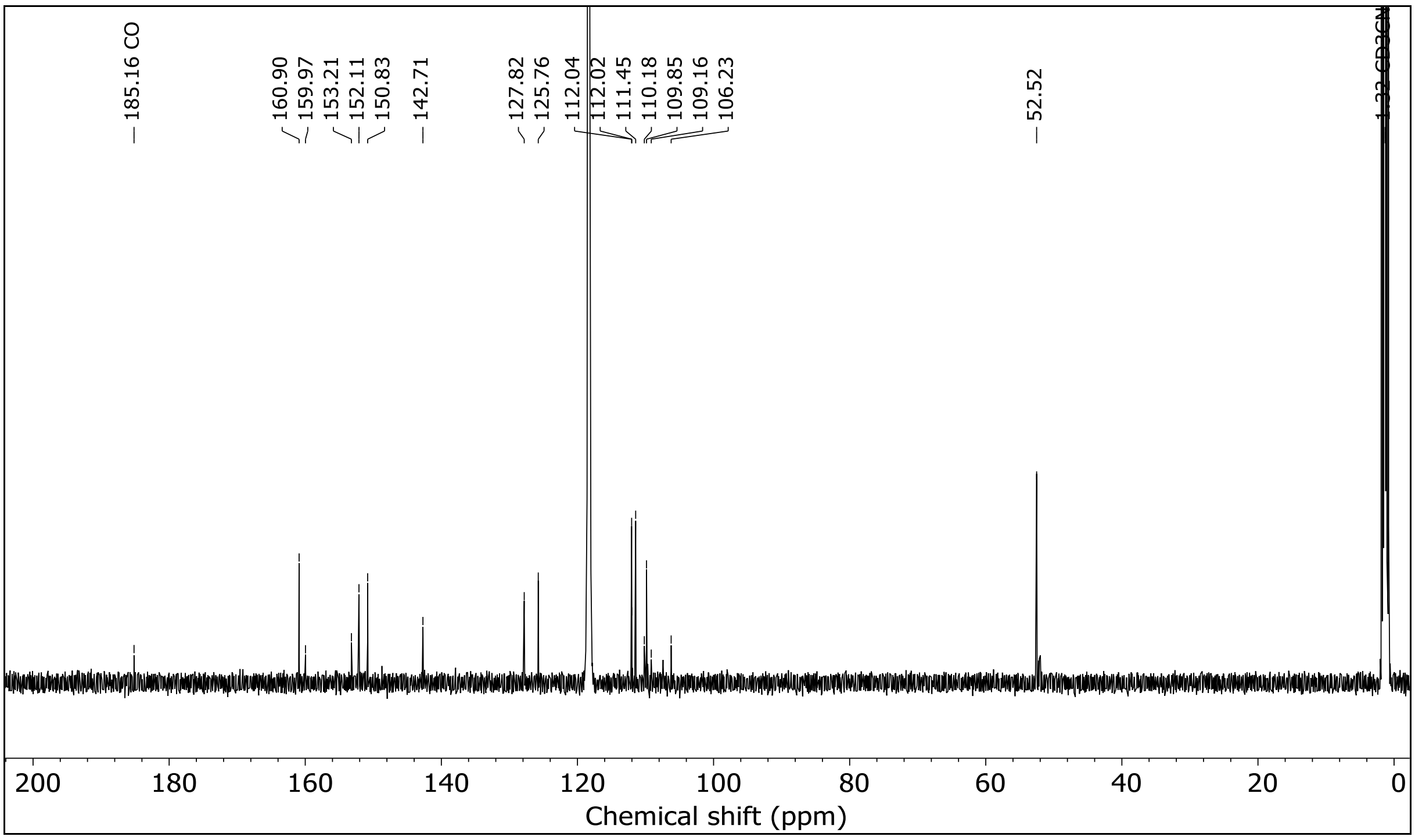

Figure S 11: ^{13}C NMR spectrum in MeCN-d3 of the reaction product of oxalyl chloride and AgCN in MeCN at low temperatures.

2. Additional pictures

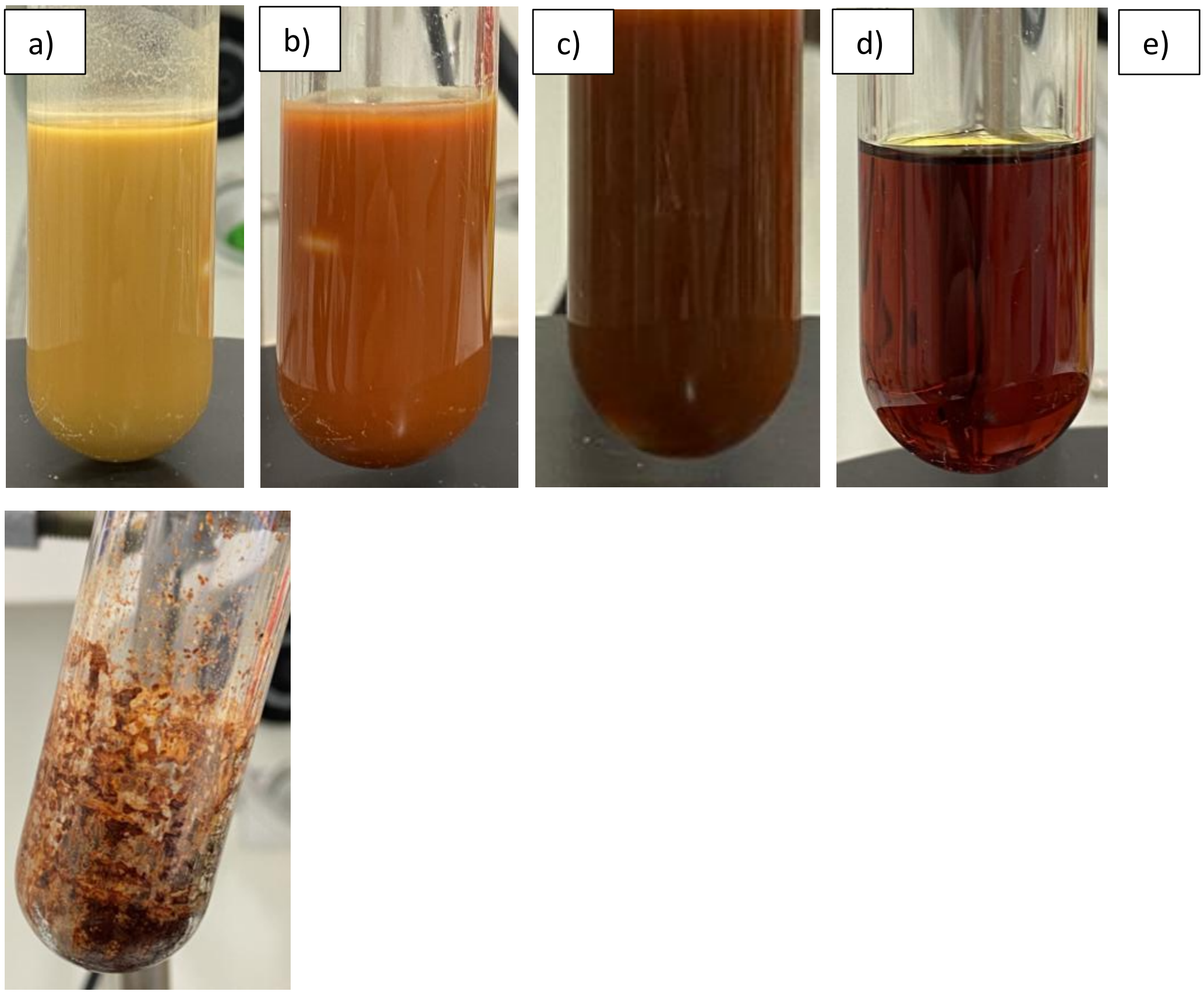

Figure S 12: Pictures of the reaction of DISN with *p*TsOH·H_2O in acetonitrile/diethyl ether at room temperature before filtration (a-c), after filtration (d) and removal of the solvent (e). For details see section "Synthesis and challenges" in the manuscript.

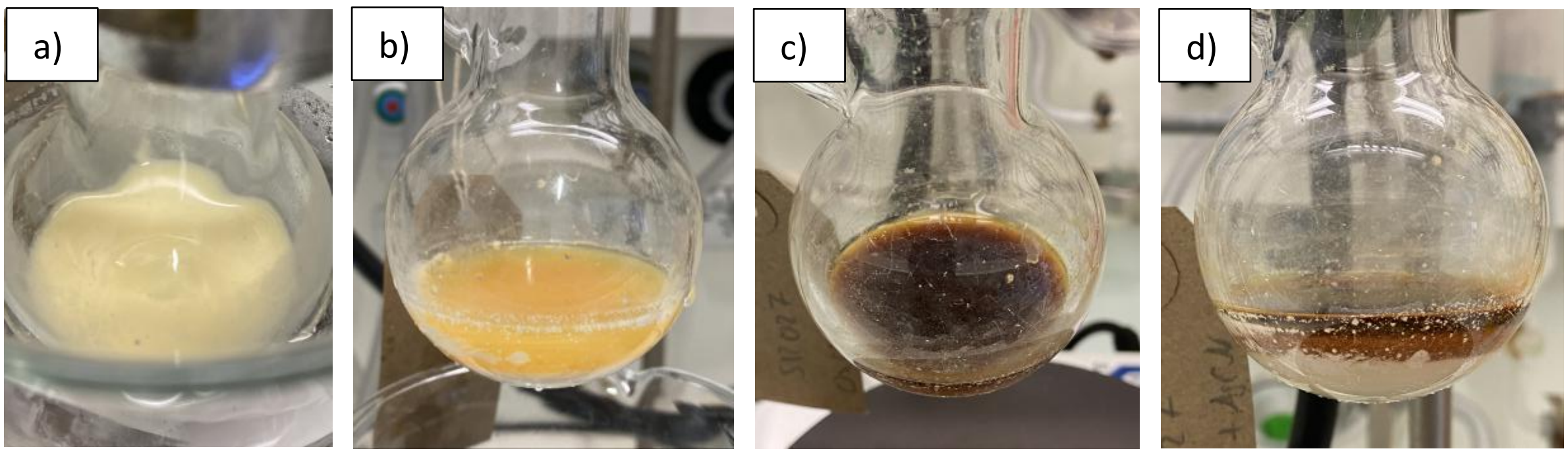

Figure S 13: Pictures of the reaction of oxalyl chloride and AgCN in acetonitrile at room temperature. For details see section "Synthesis and challenges" in the manuscript.

c) d) e)

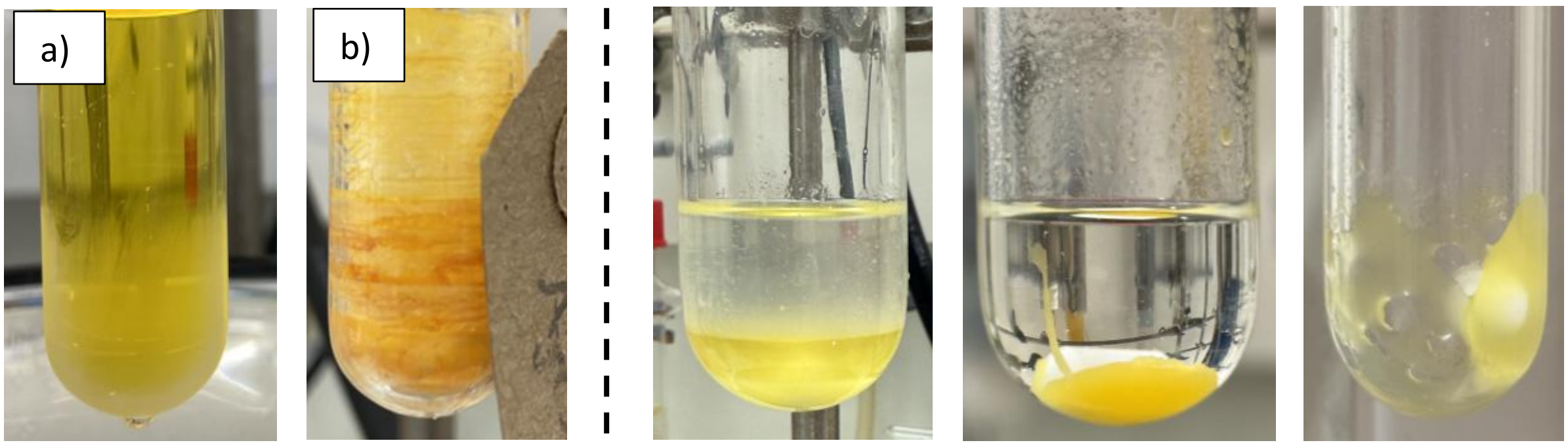

Figure S 14: Left: Pictures of the reaction of oxalyl chloride and AgCN in acetonitrile below –30 °C (a) and reaction product (b). Right: Attempts to purify the reaction product of oxalyl chloride and TMS–CN with *n*-hexane. Two phase system of *n*-hexane and TMS–Cl (c), after shaking (d) and removal of the solvent (e).

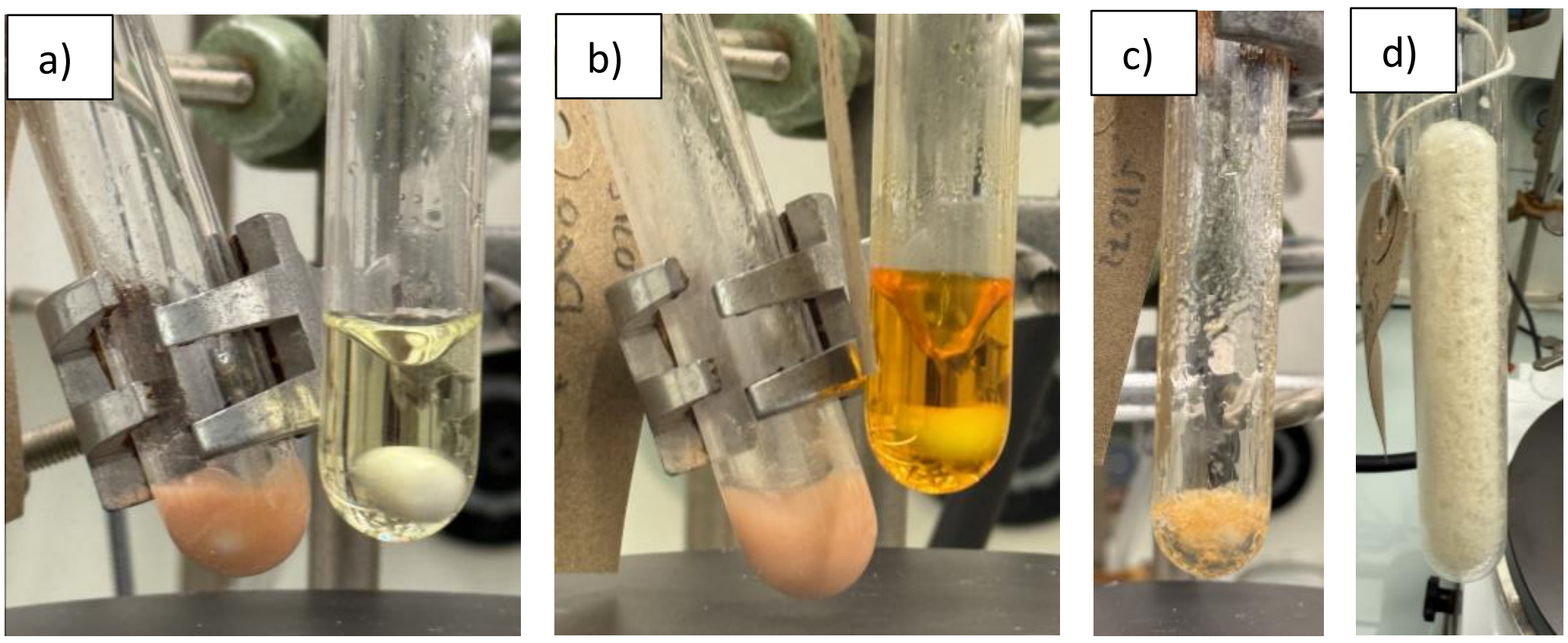

Figure S 15: Pictures of the reaction of oxalyl chloride and TMS–CN in toluene (right) and with cat. ZnI_2 in a neat reaction (left) after some time. Reaction products after removal of the solvents are shown in c and d.

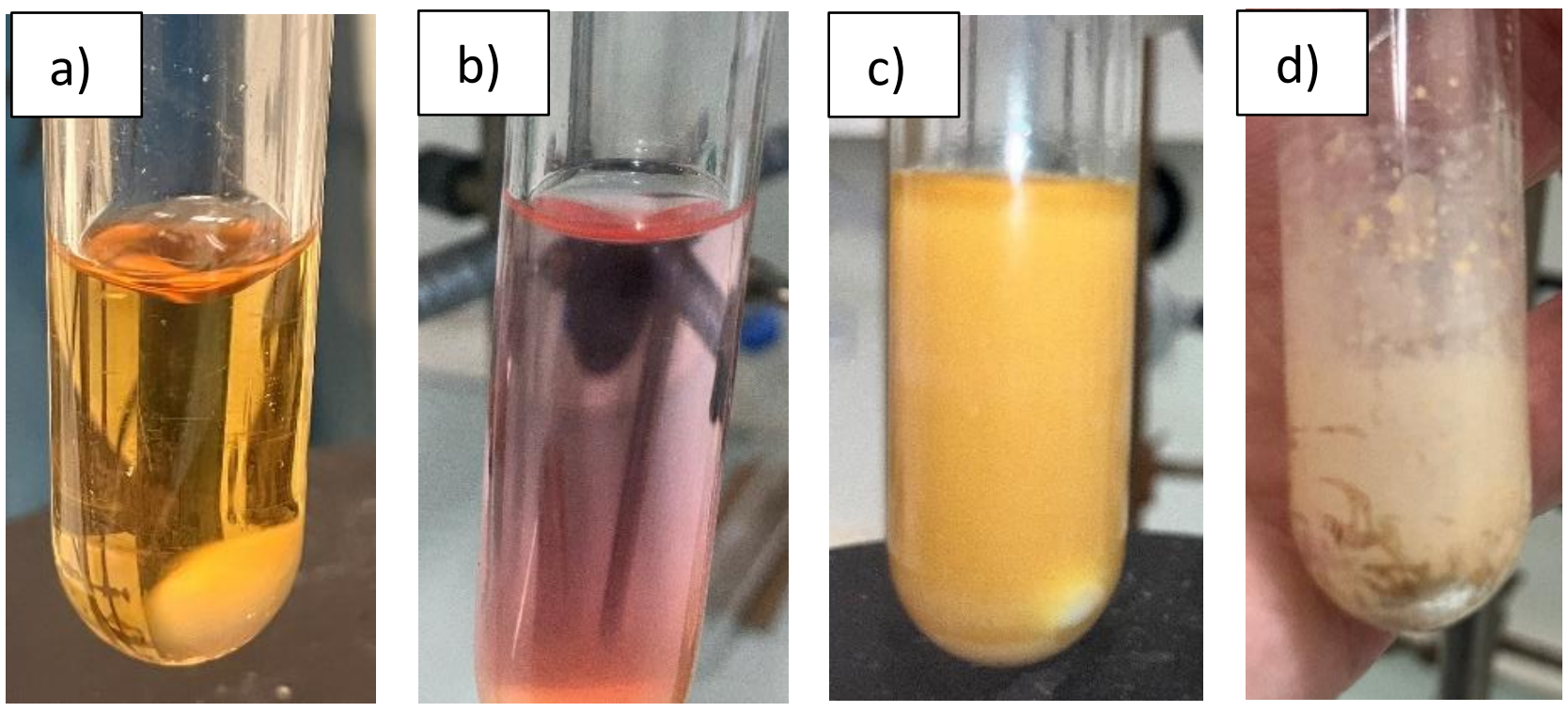

Figure S 16: Reaction of oxalyl dicyanide with CsI in DCM (a) and layered with *n*-hexane (b). Reaction with $AgBF_4$ in DCM solution (c) and after removal of solvent (d).

3. Reactivity studies

Metal complexes. All reactions were carried out either in dichloromethane at 0 °C to room temperature or in liquid SO_2 at –30 °C. Oxalyl dicyanide was reacted with one or two equivalents of the corresponding metal salts. Multiple attempts were made to synthesize metal complexes with oxalyl dicyanide, as shown for carbonyl dicyanide.[1] These reactions failed to yield an isolable product; only complex reaction mixtures were obtained. Even in the presence of extremely poorly coordinating anions such as BF_4^-, SbF_6^-, and $BaRF^-$ (tetrakis(3,5-bis(trifluoro-methyl)phenyl)borate), oxalyl dicyanide failed to form complexes with copper(I), zinc(II), silver(I), nickel(0) and caesium(I). Likewise, these reactions also failed with standard halide ligands. In the case of CsI, reduction of I^- into pink I_2 in dichloromethane solution displayed redox activity of the oxalyl dicyanide. With $AgSbF_6$, a crystalline powder was obtained after stoichiometric addition in liquid SO_2, and slow evaporation, however, this was revealed to be AgCN by powder X-ray diffraction. Similar, the reaction of oxalyl dicyanide with $AgBF_4$ yielded a pale brown product. PXRD analysis indicated the consumption of the starting material, but also the formation of AgCl and AgCN as the dominant phases, in addition to an amorphous background. On the other hand, the corresponding IR spectrum (see Figure S 17) might show signals, which suggest the formation of the desired product. In general, these reactions show the severe lability of oxalyl dicyanide and its ineffectiveness as a ligand at the current state and needs further research.

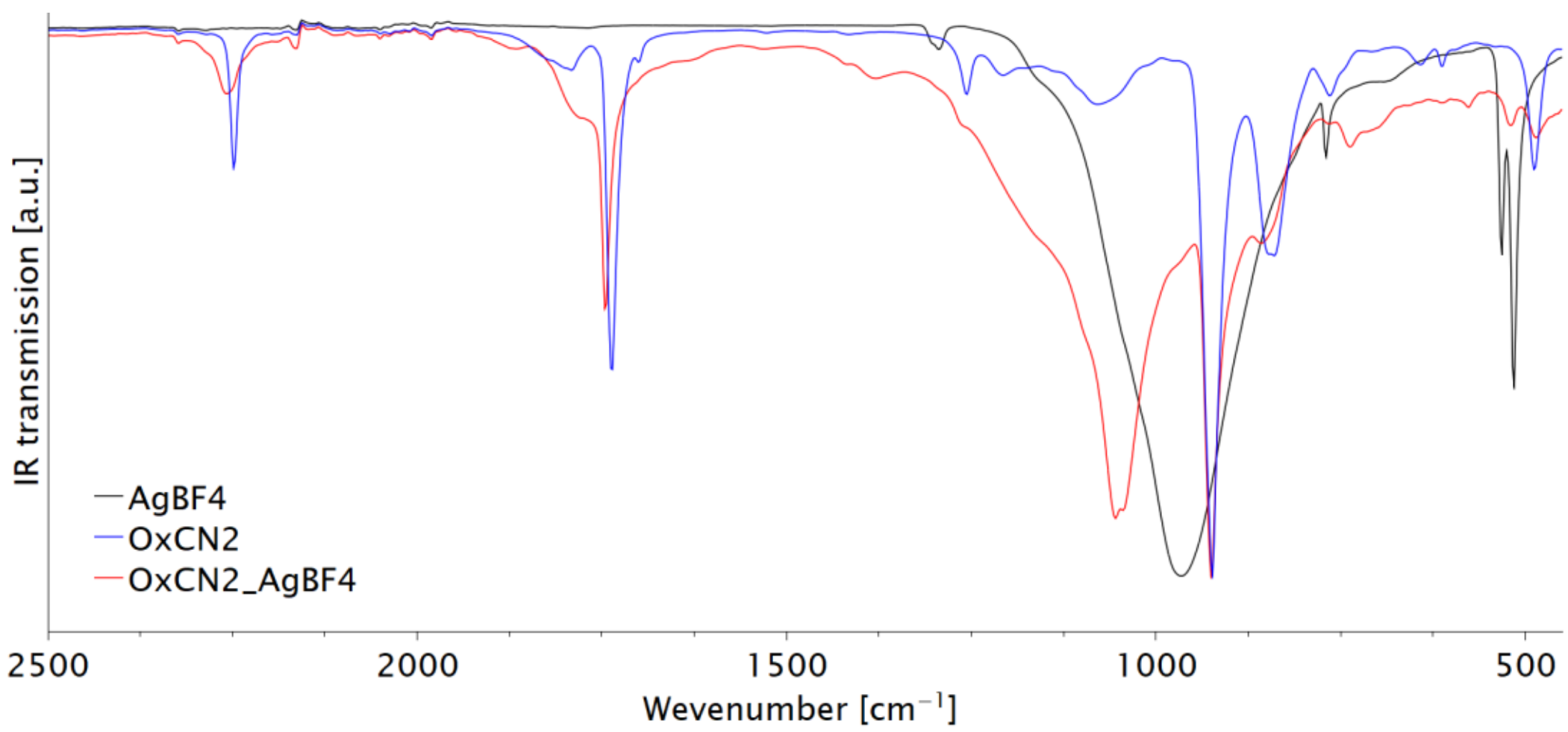

Figure S 17: IR spectrum of $AgBF_4$, oxalyl dicyanide and the reaction of both.

Nucleophilic substitution: Similar to the brief description of its reactivity towards amines, we were interested in, whether the reaction of oxalyl dicyanide with ethanol would result in a

[1] A. Decken, C. Knapp, J. Passmore, *J. Chem. Cryst.* **2005**, 35, 697-700.

similar pattern. Multiple attempts were made; however, undefinable greasy products were obtained. NMR spectroscopy indicated the formation of the corresponding diethyl oxalate (^{1}H: 4.38, 1.39 ppm; ^{13}C: 63.5, 14.0 ppm in $CDCl_3$) in comparison with literature data (^{1}H: 4.36, 1.38 ppm; ^{13}C: 158.1, 63.0, 13.9 ppm). The signals were also significantly broadened and smeared, suggesting the presence of polymeric species.

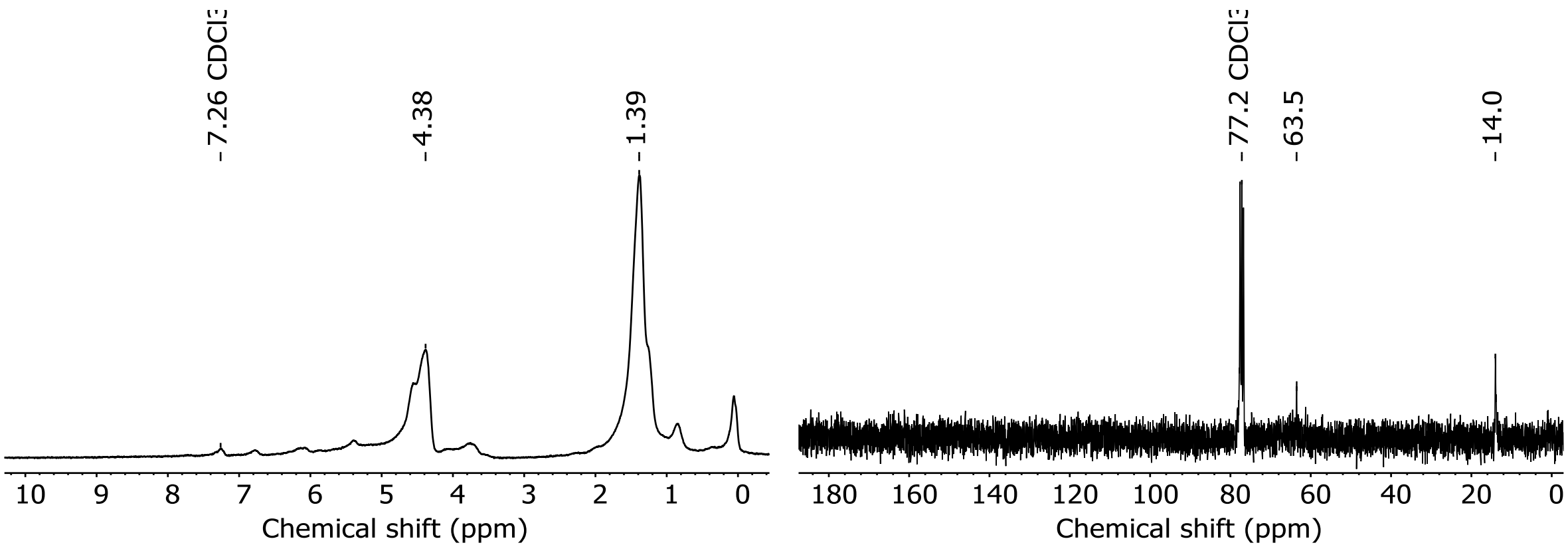

Figure S 18: ^{1}H (left) and ^{13}C (right) NMR spectrum in $CDCl_3$ of the reaction product of oxalyl dicyanide with two equivalents of ethanol.

Controlled hydrolyses. Two attempts were made to obtain 2,2,3,3-tetrahydroxy-succinonitrile. 1) TMS-CN (0.31 mL, 0.25 mmol) was added dropwise to $(COCl)_2$ (0.43 mL, 5.00 mmol) at room temperature. After removal of all volatile components, the Schlenk flask was opened to air for two hours, yielding a sticky oil. The infrared spectrum showed no evidence of the formation of the desired compound, but a characteristic vibrational band for a carbonyl group, similar to that of oxalic acid. 2) Oxalyl dicyanide (63.4 mg, 0.587 mmol) was dissolved in 5 mL DCM and water (0.0212 mg, 1.17 mmol) was added to form orange droplets. After removal of the solvent a yellow oil was obtained, which was not further characterized.

4. Crystallographic data

Growth of single crystals of oxalyl dicyanide: Single crystals were grown and selected directly from the reaction flask.

Growth of single crystals of 2,2,3,3-tetrahydroxysuccinonitrile: Single crystals were grown by hydrolysis in Paratone-N oil on the microscope slide, while selecting crystals of oxalyl dicyanide.

Growth of single crystals of the 1,4-dioxane oxalyl dicyanide complex: Single crystals were grown and selected directly from the reaction flask.

Table S 1: Selected single crystal X-ray data collection and refinement parameters for oxalyl dicyanide (**1**), 1,4-dioxane oxalyl dicyanide complex (**2**) and 2,2,3,3-tetrahydroxysuccinonitrile (**3**).

	**1**	**2**	**3**
Formula	$C_4N_2O_2$	$C_8H_8N_2O_4$	$C_4H_4N_2O_4$
CCDC			
F. w. / g mol^{-1}	108.06	196.16	144.09
Crystal system	orthorhombic	triclinic	monoclinic
Space group	*Cmce*	$P\bar{1}$	$P2_1/n$
a / Å	7.0770(9)	5.6902(6)	7.1094(3)
b / Å	7.5273(12)	6.8610(7)	6.2431(2)
c / Å	8.7580(10)	7.1350(7)	7.3185(3)
α / °	90	111.665(7)	90
β / °	90	112.134(7)	111.219(3)
γ / °	90	94.089(8)	90
V / Å^3	466.54(11)	232.53(4)	302.81(2)
Z	4	1	2
Radiation, λ / Å	1.54186	1.54186	1.54186
Temp / K	100	100	100
ρ_{calc} / g cm^{-3}	1.538	1.401	1.580
μ / mm^{-1}	1.126	0.983	1.271
Reflections collected	2281	5455	5679
Ind. Reflns. / Ind. Reflns gt	258 / 190	832 / 734	607 / 549
Parameters	30	74	54
R_{int} / $R_{(\sigma)}$ / %	0.0174 / 0.0373	0.0080 / 0.0145	0.0114 / 0.0254
*R*1/*wR*2,[a] $I \geq 2\sigma I$ / %	2.99 / 7.55	4.00 / 10.08	3.00 / 7.60
*R*1/*wR*2,[a] all data / %	4.42 / 7.89	4.53 / 10.52	3.41 / 7.83
GOF	0.962	1.060	1.071
Twin law	—	—	—
BASF	—	—	—
Flack parameter	—	—	—

[a] $R1 = [\Sigma||F_o| - |F_c||]/\Sigma|F_o|$; $wR2 = \{[\Sigma w[(F_o)^2 - (F_c)^2]^2]/[\Sigma w(F_o^2)^2\}^{1/2}$; $w = [\sigma^2(F_o)^2 + (AP)^2 + BP]^{-1}$, where $P = [(F_o)^2 + 2(F_c)^2]/3$ and the A and B values are 0.052400 and 0.000 for **1**, 0.046700 and 0.143900 for **2**, and 0.046800 and 0.088900 for **3**.

4.1. Additional Pictures for oxalyl dicyanide

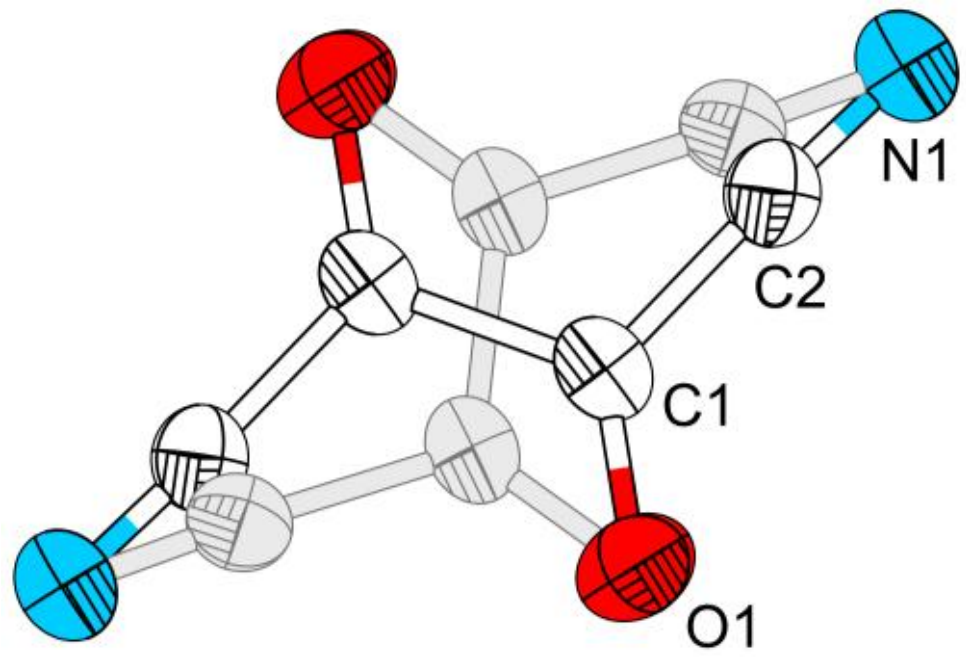

Figure S 19: Molecular structure of oxalyl dicyanide in the single crystal. Ellipsoids are drawn with 70% displacement at 100 K. Color code: O red, C white and N blue.

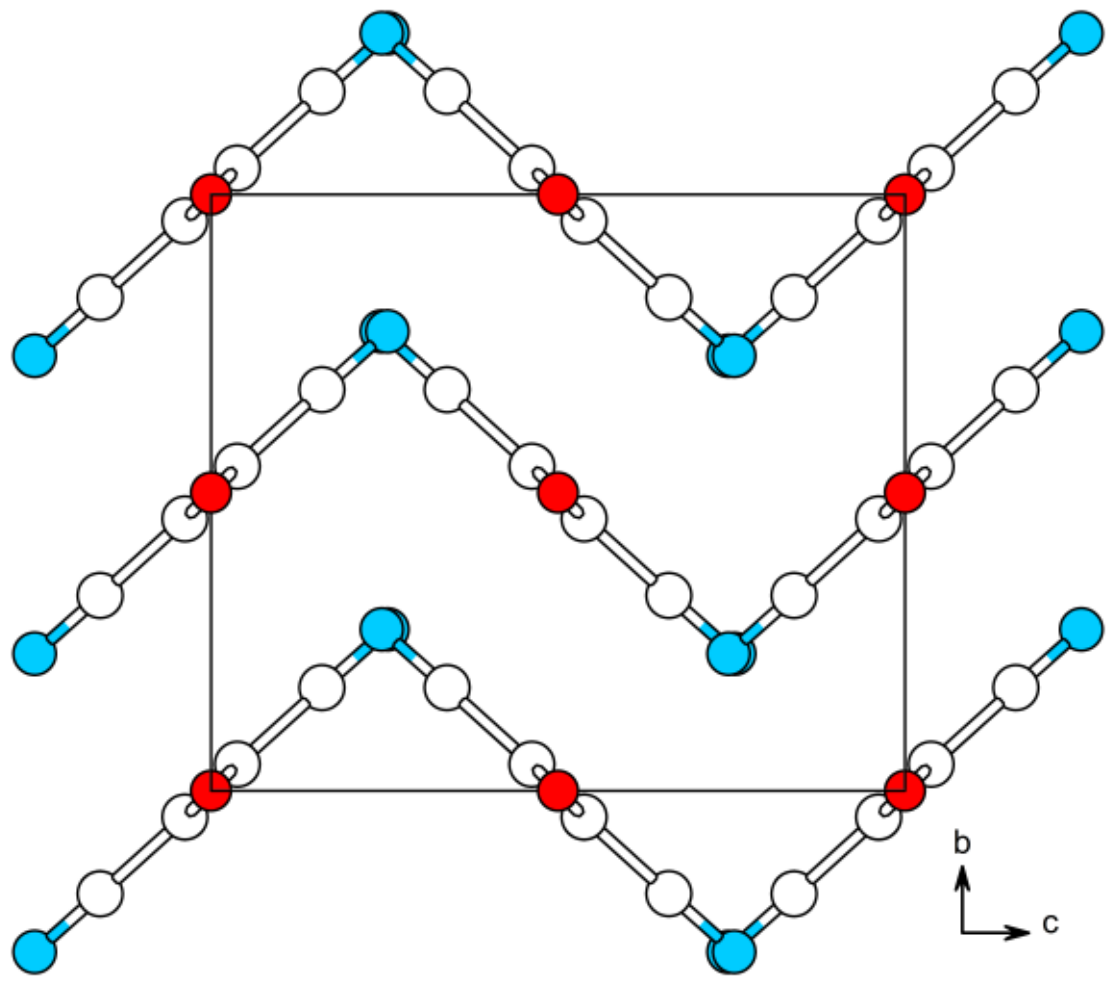

Figure S 20: Crystal structure of oxalyl dicyanide viewed along [100]. Atoms are depicted with arbitrary radii. Color code: O red, C white and N blue.

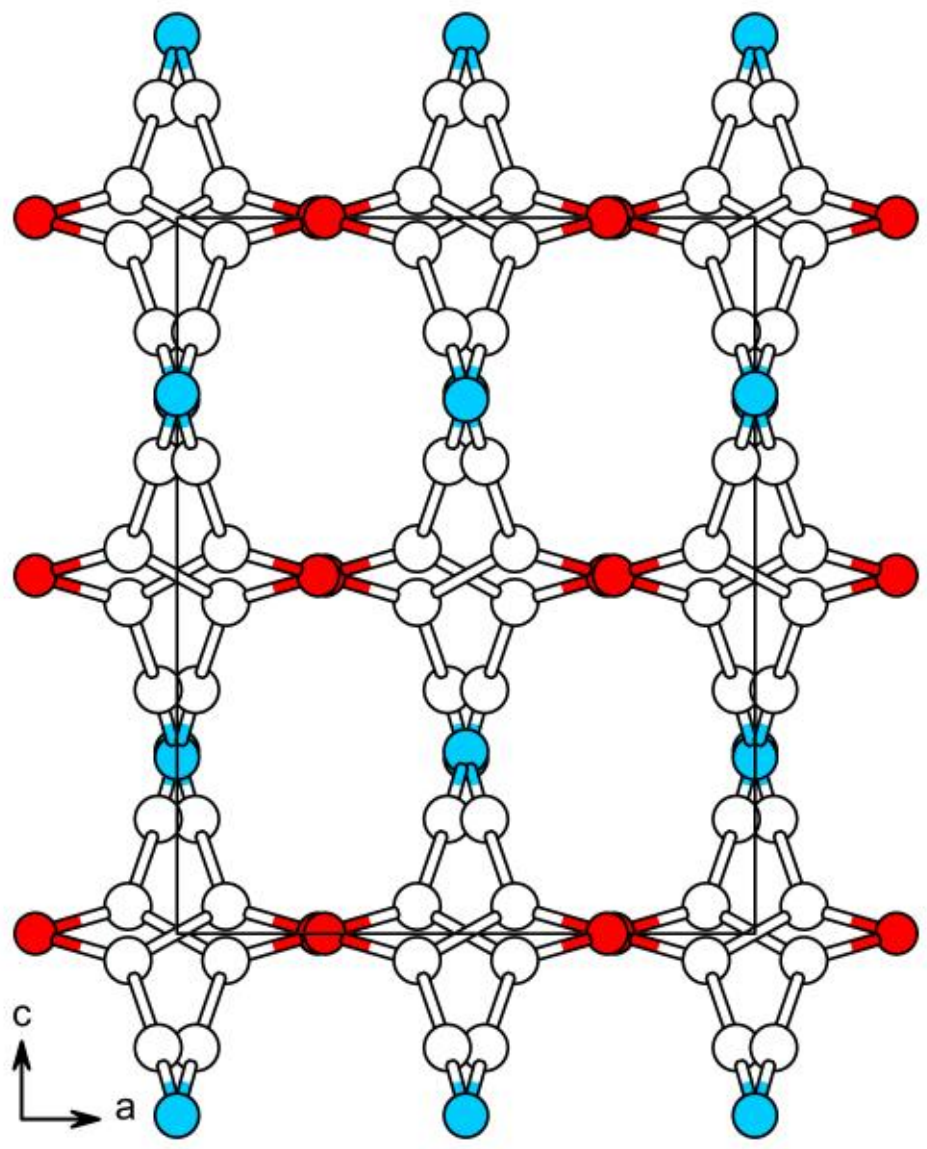

Figure S 21: Crystal structure of oxalyl dicyanide viewed along [010]. Atoms are depicted with arbitrary radii. Color code: O red, C white and N blue.

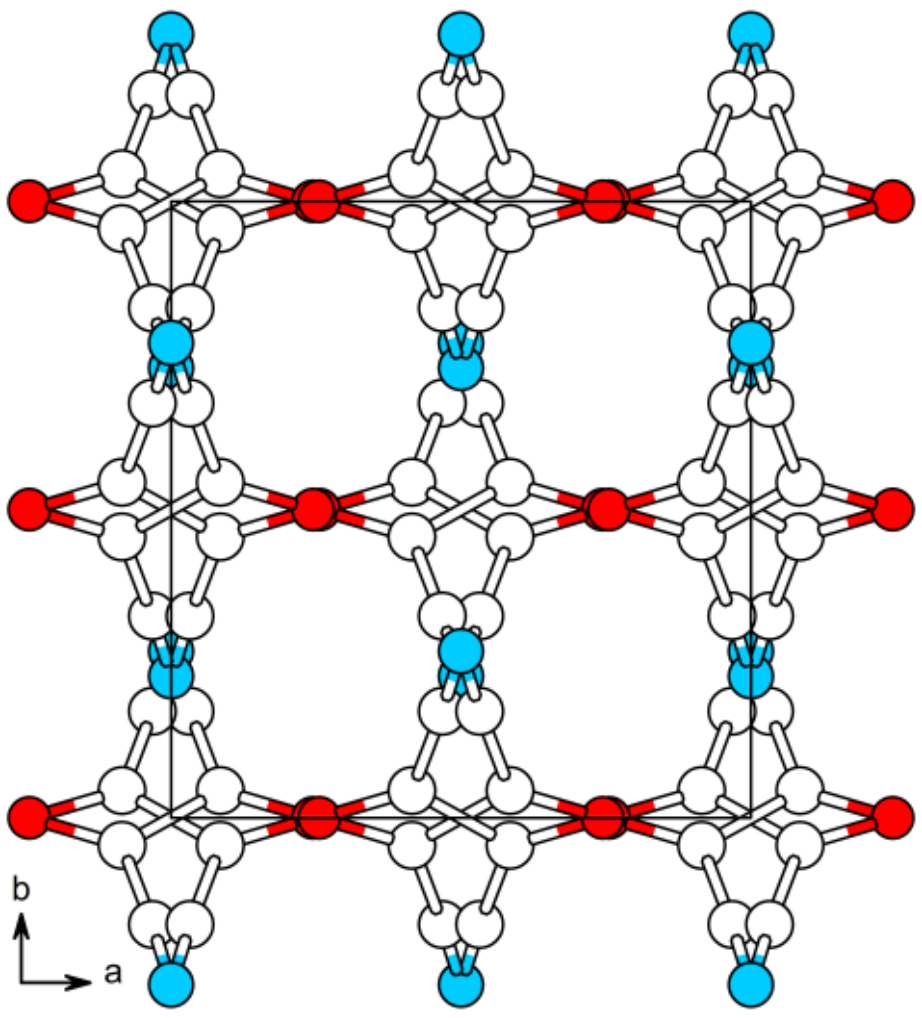

Figure S 22: Crystal structure of oxalyl dicyanide viewed along [001]. Atoms are depicted with arbitrary radii. Color code: O red, C white and N blue.

4.2. Additional Pictures for the 1,4-dioxane oxalyl dicyanide complex

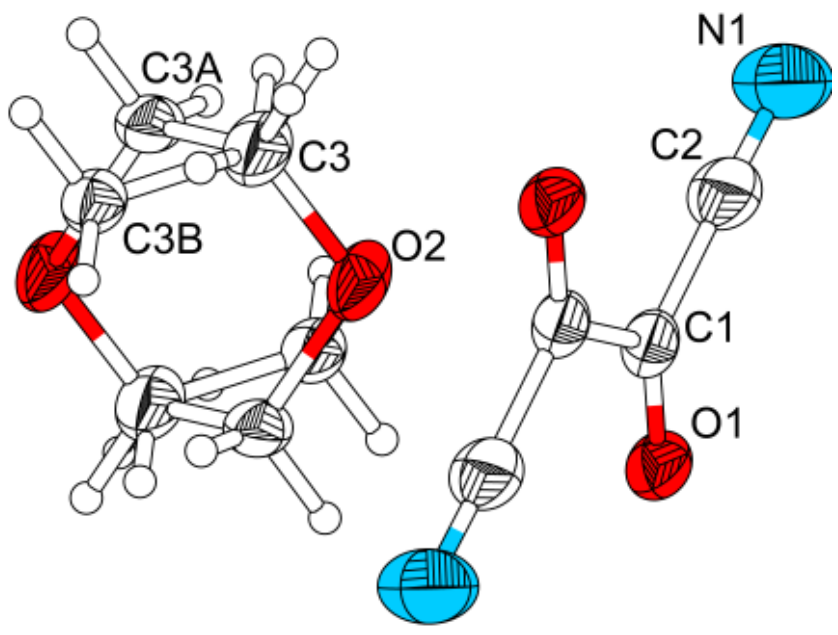

Figure S 23: Molecular structure of the 1,4-dioxane oxalyl dicyanide complex in the single crystal. Ellipsoids are drawn with 60% displacement at 100 K. Color code: O red, C white and N blue.

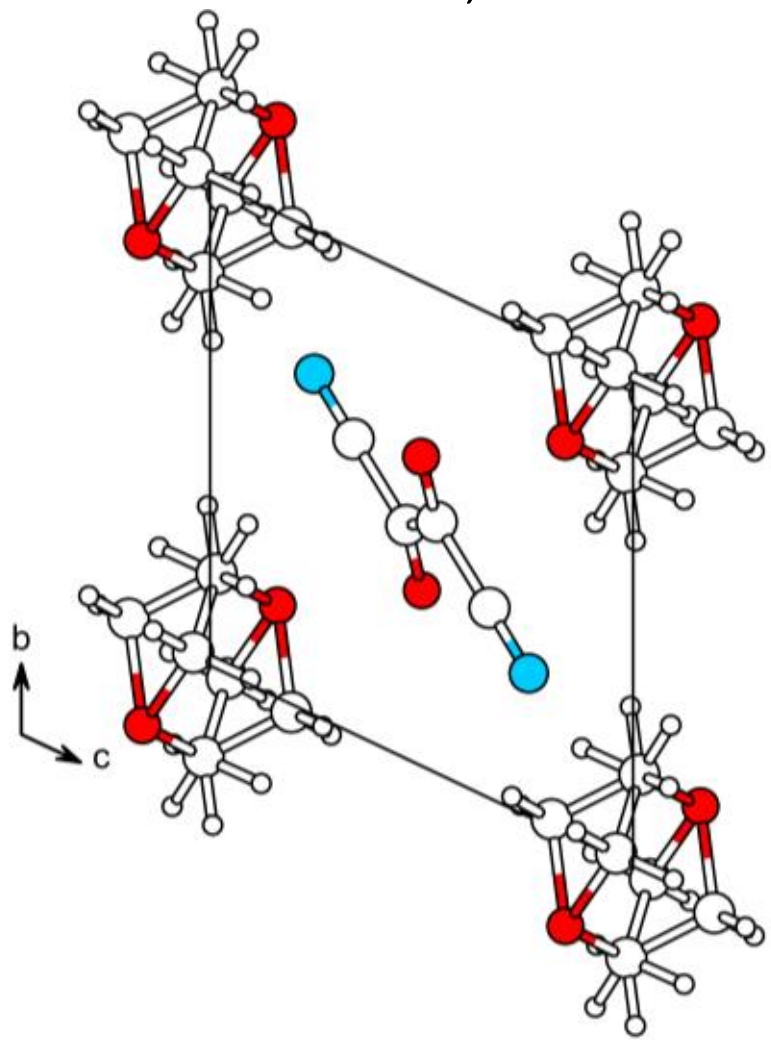

Figure S 24: Crystal structure of the 1,4-dioxane oxalyl dicyanide complex viewed along [100]. Atoms are depicted with arbitrary radii. Color code: O red, C white and N blue.

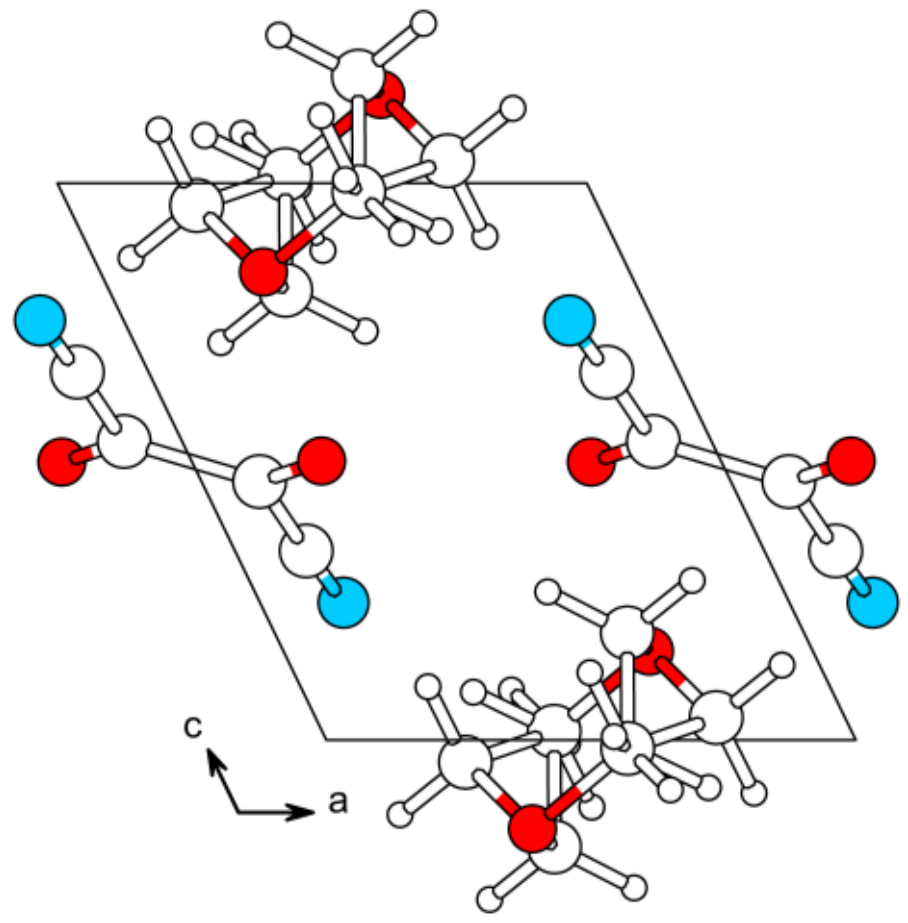

Figure S 25: Crystal structure of the 1,4-dioxane oxalyl dicyanide complex viewed along [010]. Atoms are depicted with arbitrary radii. Color code: O red, C white and N blue.

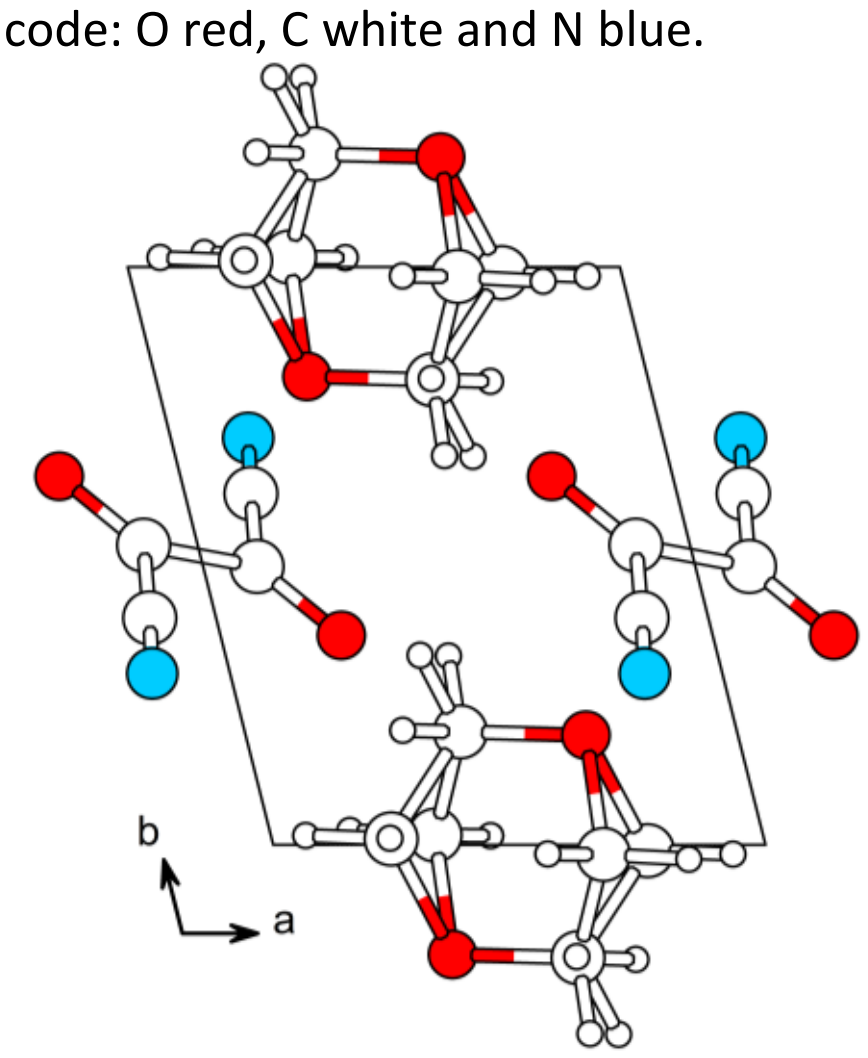

Figure S 26: Crystal structure of 1,4-dioxane oxalyl dicyanide viewed along [001]. Atoms are depicted with arbitrary radii. Color code: O red, C white and N blue.

4.3. Additional Pictures for 2,2,3,3-tetrahydroxysuccinonitrile

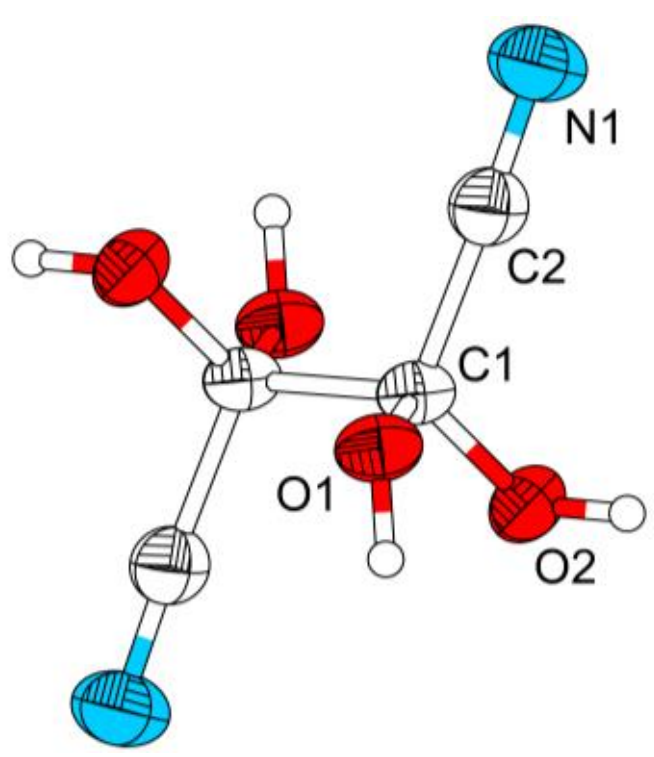

Figure S 27: Molecular structure of 2,2,3,3-tetrahydroxysuccinonitrile in the single crystal. Ellipsoids are drawn with 80% displacement at 100 K. Color code: O red, C white and N blue.

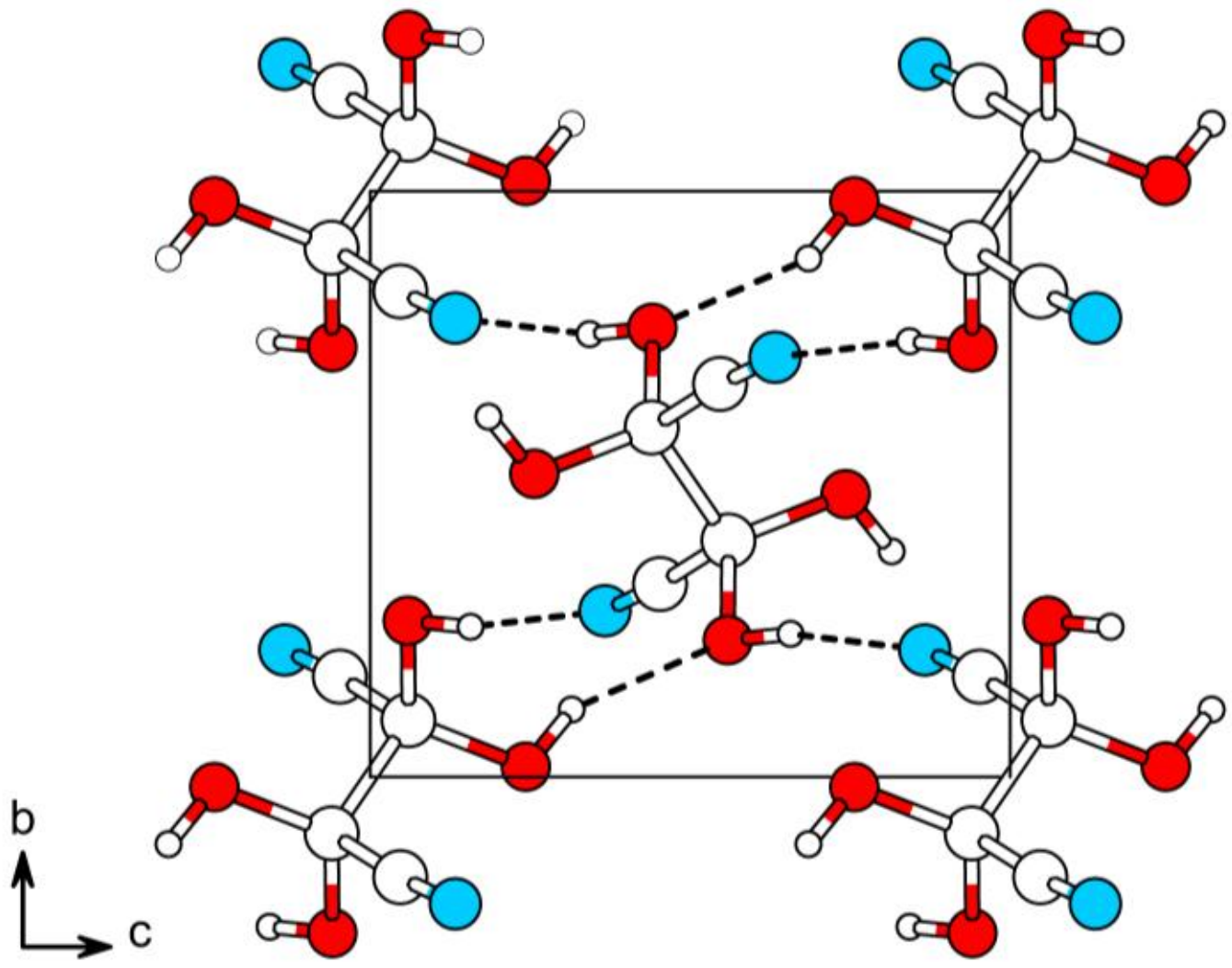

Figure S 28: Crystal structure of 2,2,3,3-tetrahydroxysuccinonitrile viewed along [100]. Atoms are depicted with arbitrary radii. Color code: O red, C white and N blue.

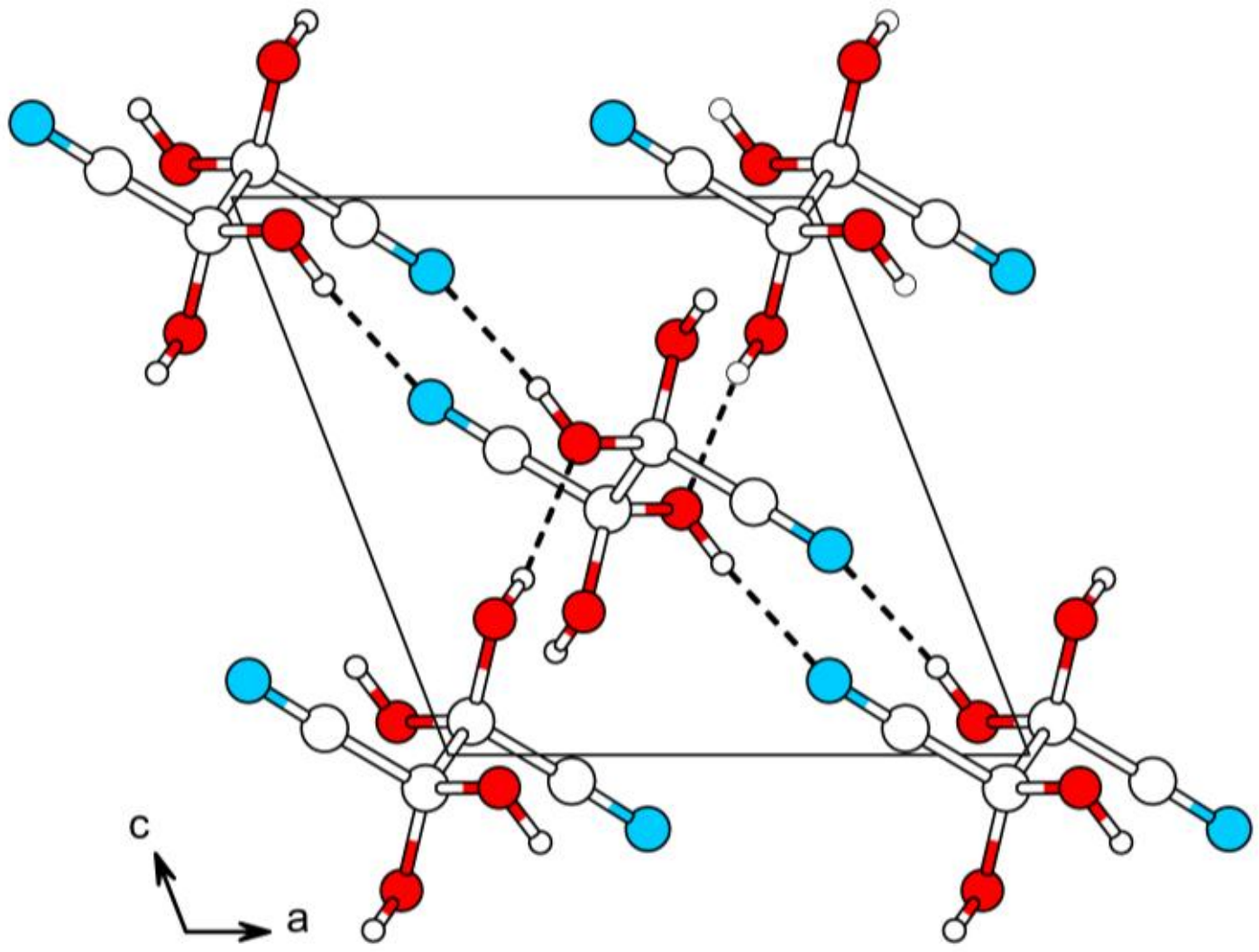

Figure S 29: Crystal structure of 2,2,3,3-tetrahydroxysuccinonitrile viewed along [010]. Atoms are depicted with arbitrary radii. Color code: O red, C white and N blue.

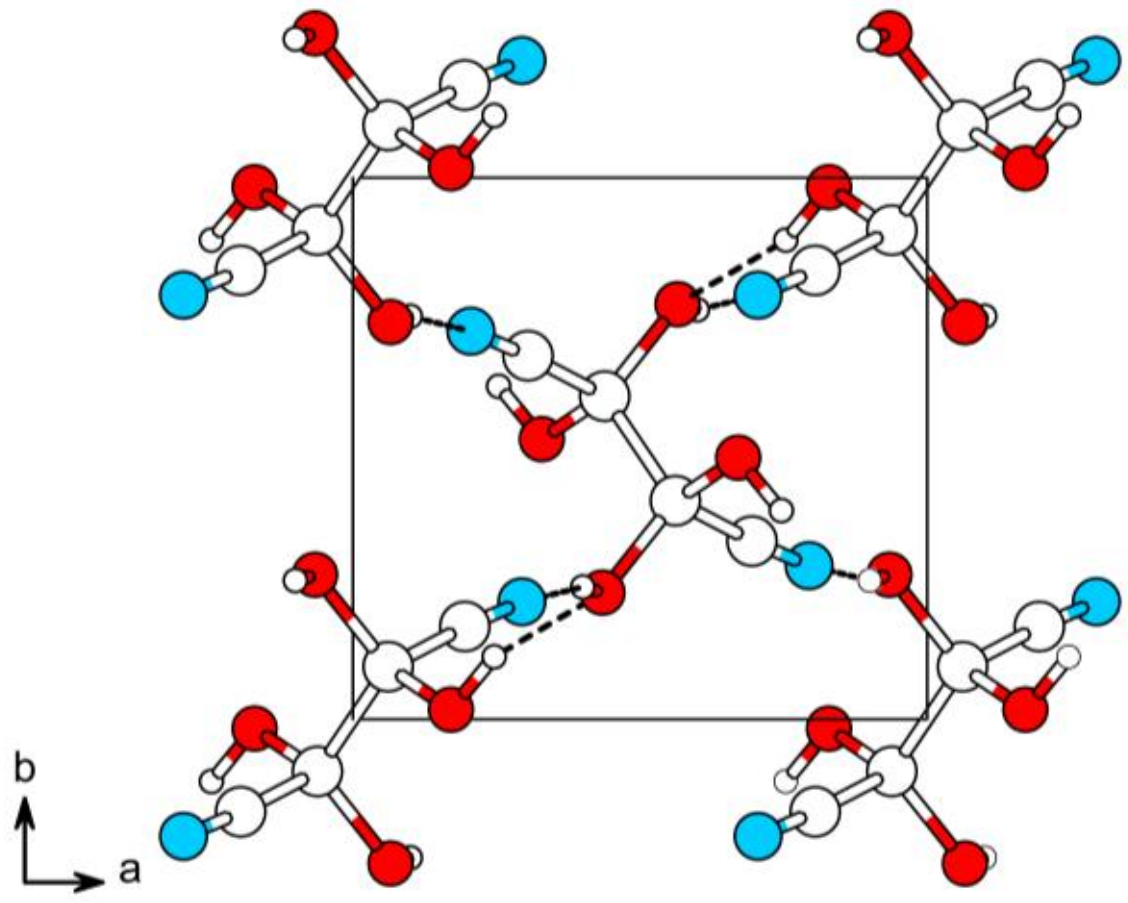

Figure S 30: Crystal structure of 2,2,3,3-tetrahydroxysuccinonitrile viewed along [001]. Atoms are depicted with arbitrary radii. Color code: O red, C white and N blue.

5. Additional details on Hirshfeld surface analysis

5.1. Oxalyl dicyanide

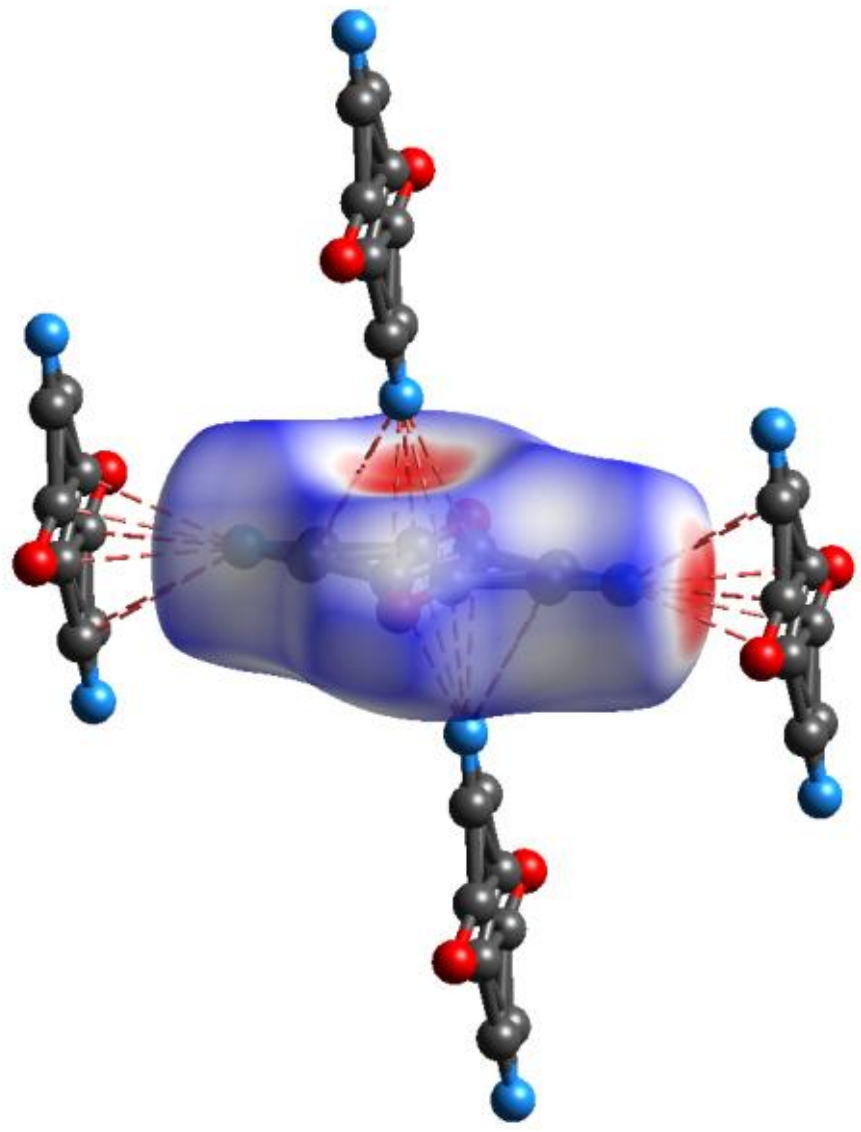

Figure S 31: Hirshfeld surface of oxalyl dicyanide at 100 K shown with neighboring molecules. Red areas indicate short contacts. Red dashed lines highlight C···N short contacts. Color code: C grey, O red and N blue.

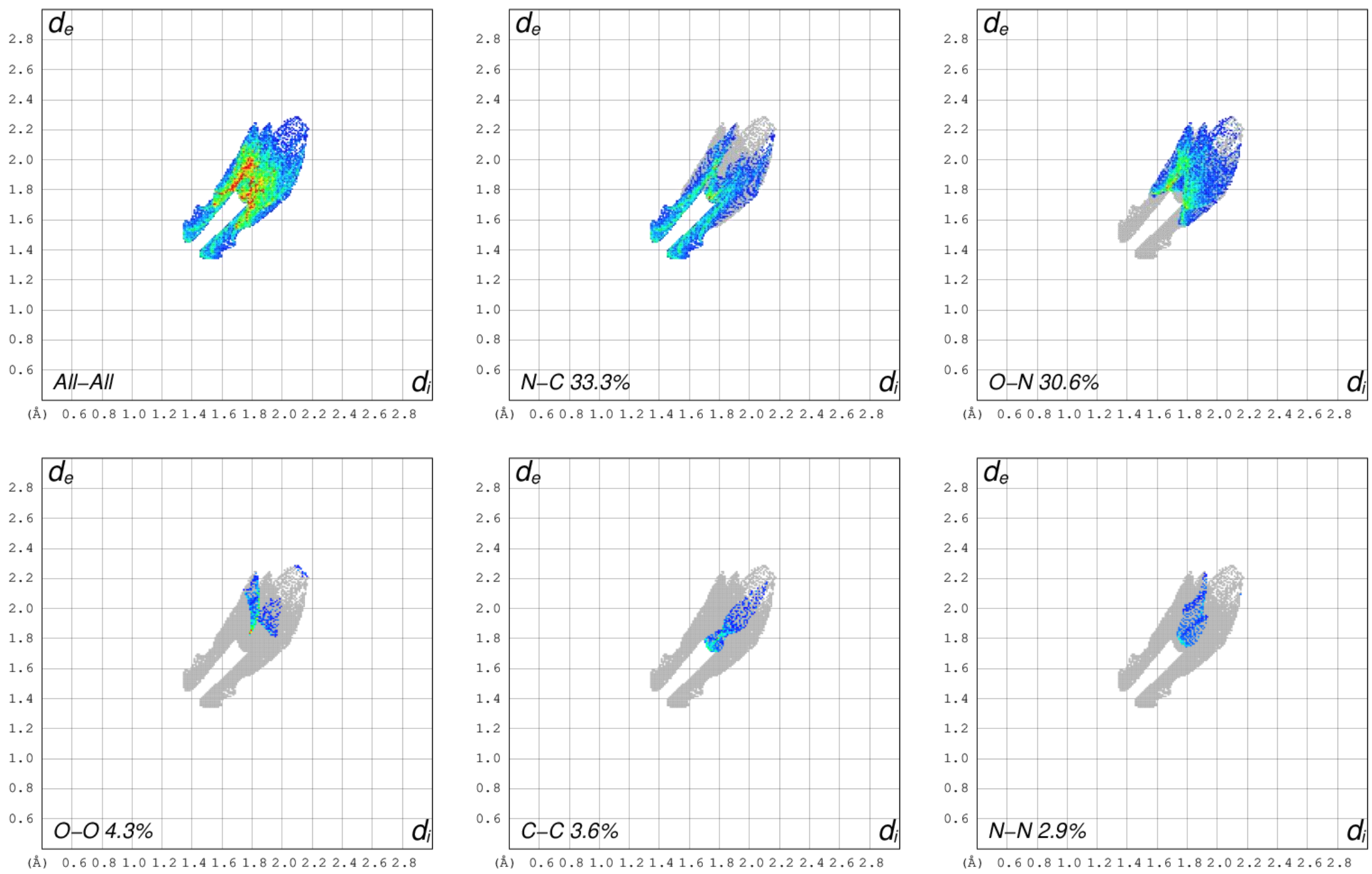

Figure S 32: Fingerprint plots for oxalyl dicyanide at 100 K mapped from the Hirshfeld surface. Surface contacts and overall quantity are provided in each graph.

5.2. 1,4-Dioxane oxalyl dicyanide complex

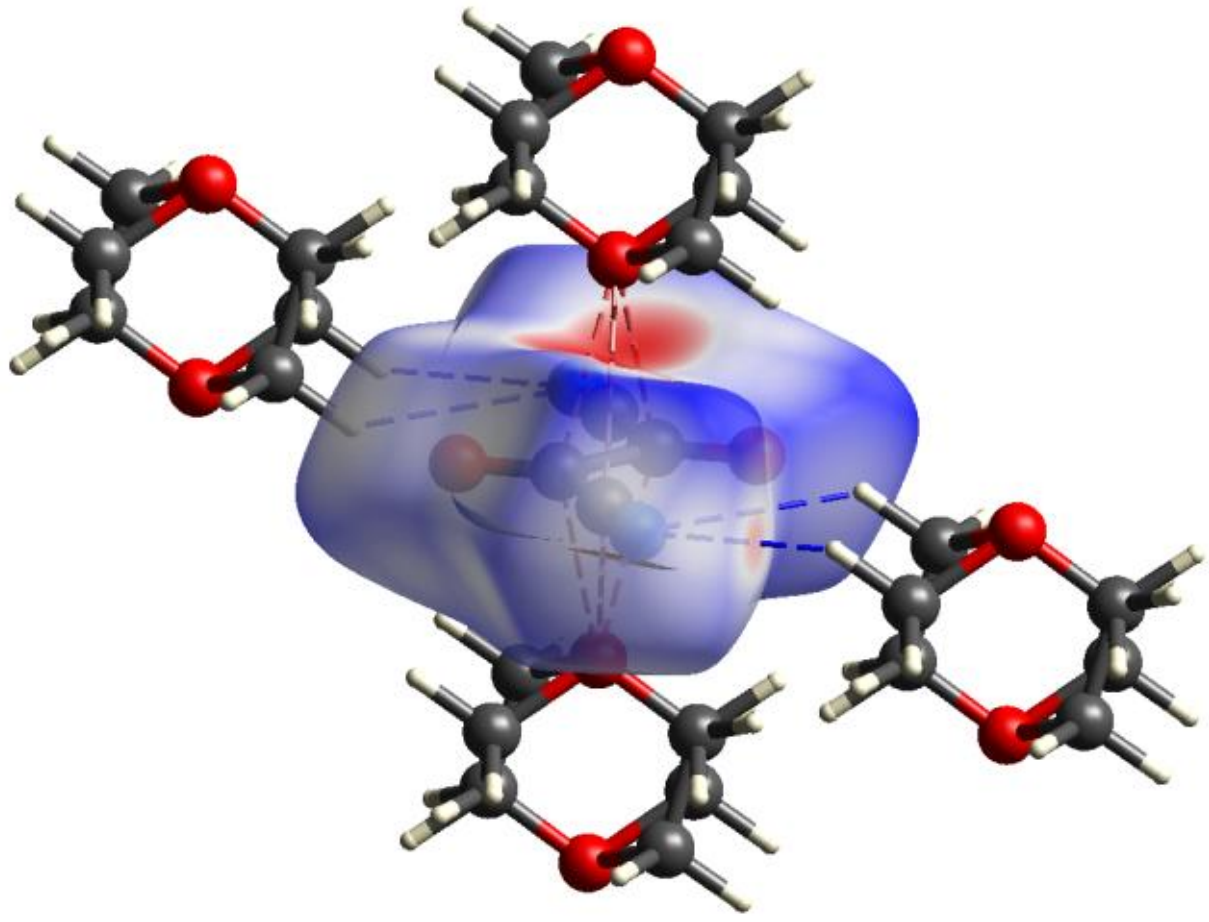

Figure S 33: Hirshfeld surface of the 1,4-dioxane oxalyl dicyanide complex at 100 K shown with neighboring molecules. Red areas indicate short contacts. Red dashed lines highlight C···O short contacts and blue dashed lines highlight N···H short contacts. Color code: C grey, O red, N blue and H white.

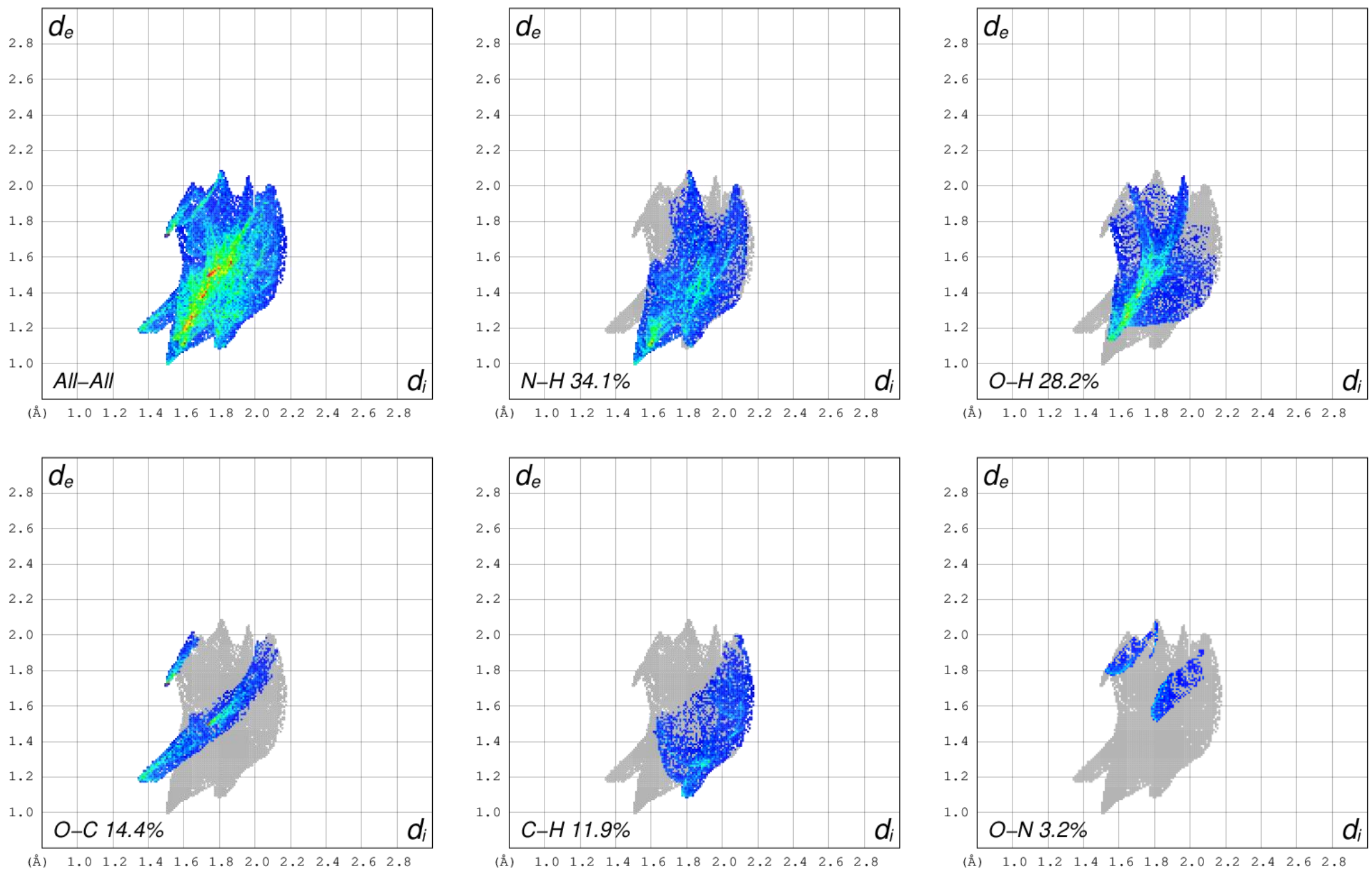

Figure S 34: Fingerprint plots for the 1,4-dioxane oxalyl dicyanide complex at 100 K mapped from the Hirshfeld surface. Surface contacts and overall quantity are provided in each graph.

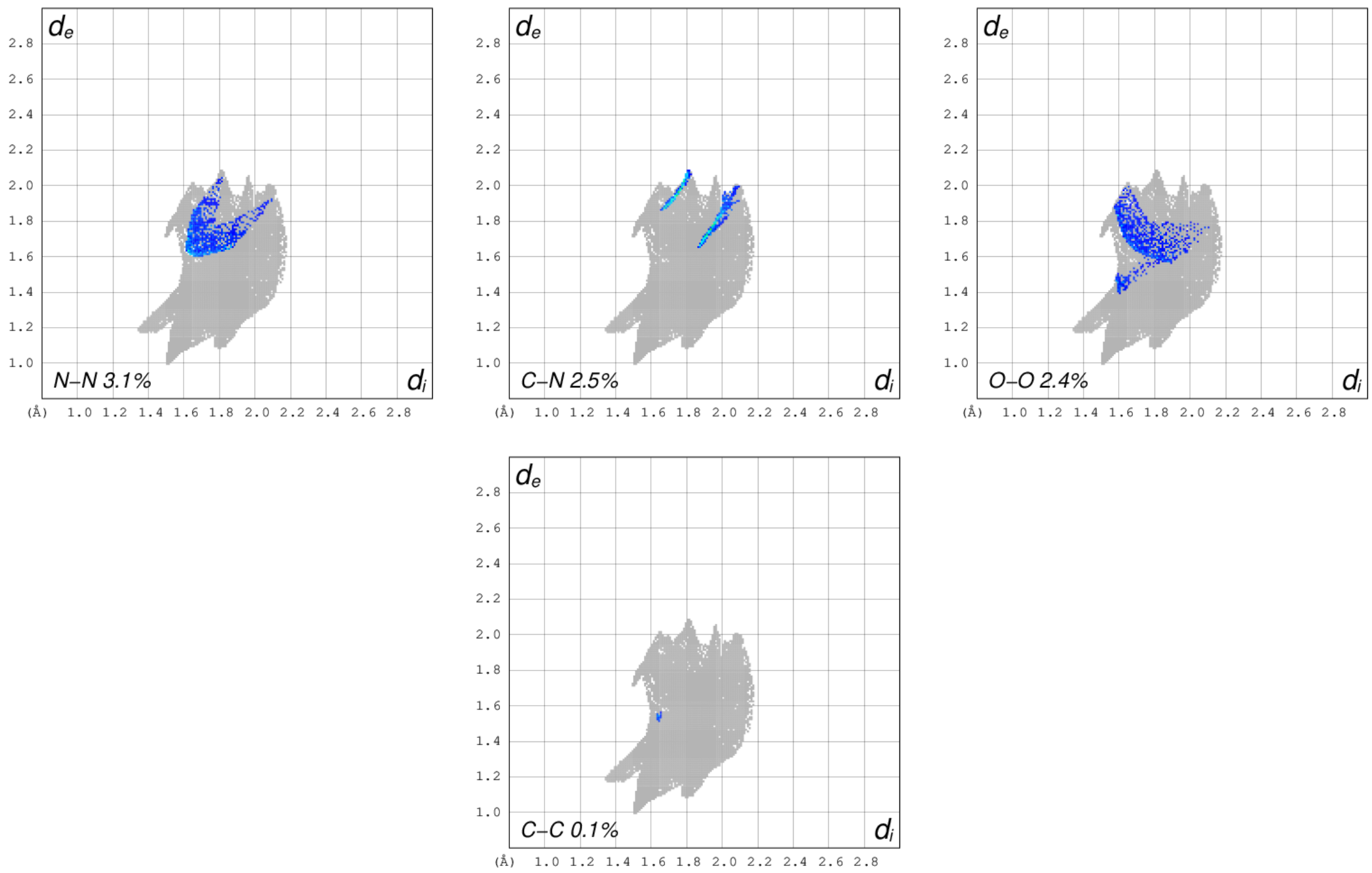

Figure S 35 continued: Fingerprint plots for the 1,4-dioxane oxalyl dicyanide complex at 100 K mapped from the Hirshfeld surface. Surface contacts and overall quantity are provided in each graph.

5.3. 2,2,3,3-Tetrahydroxysuccinonitrile

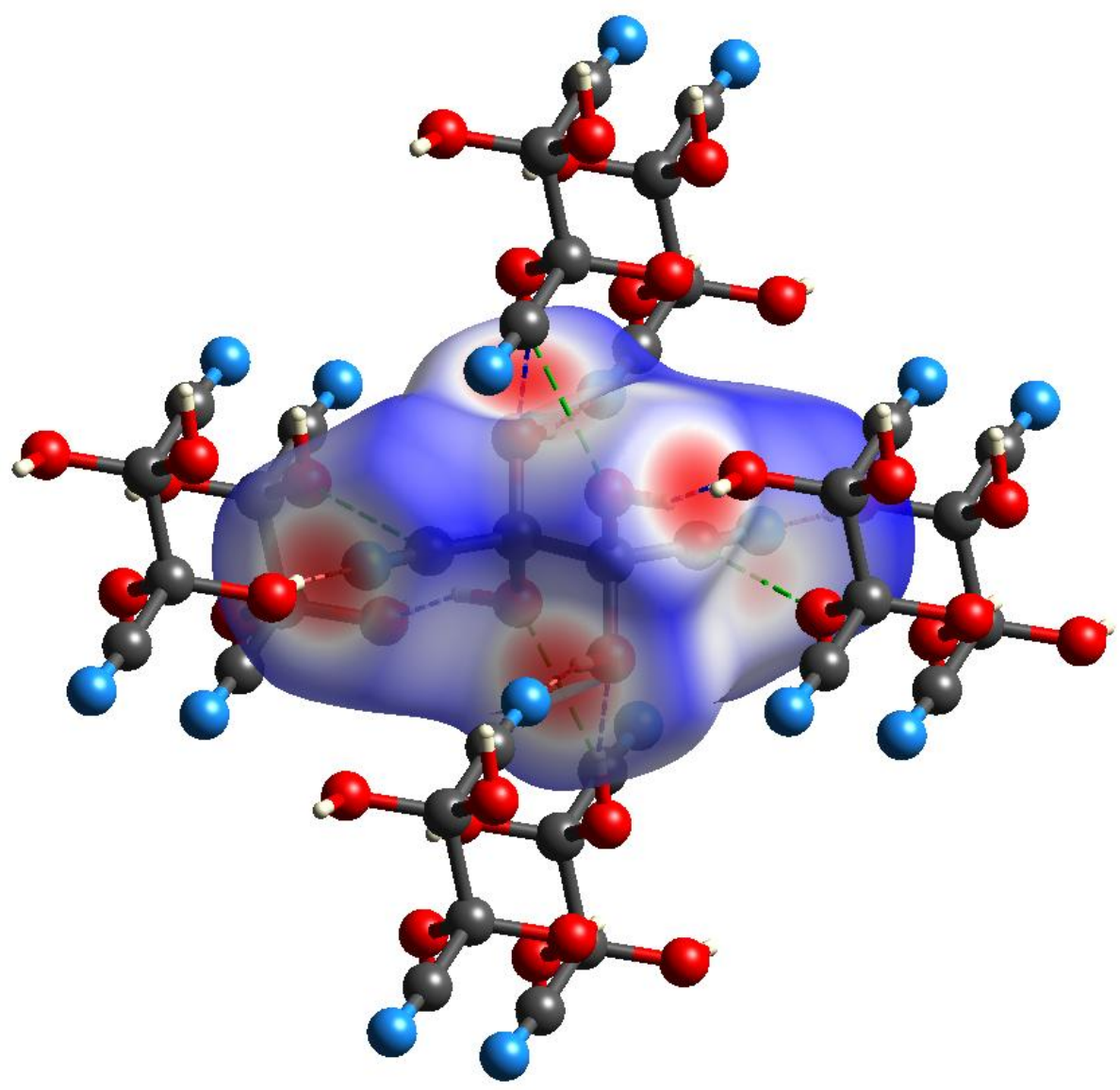

Figure S 36: Hirshfeld surface of 2,2,3,3-tetrahydroxysuccinonitrile at 100 K shown with neighboring molecules. Red areas indicate short contacts. Blue dashed lines highlight O⋯H short contacts and green dashed lines highlight O⋯C short contacts. Color code: C grey, O red, N blue and H white.

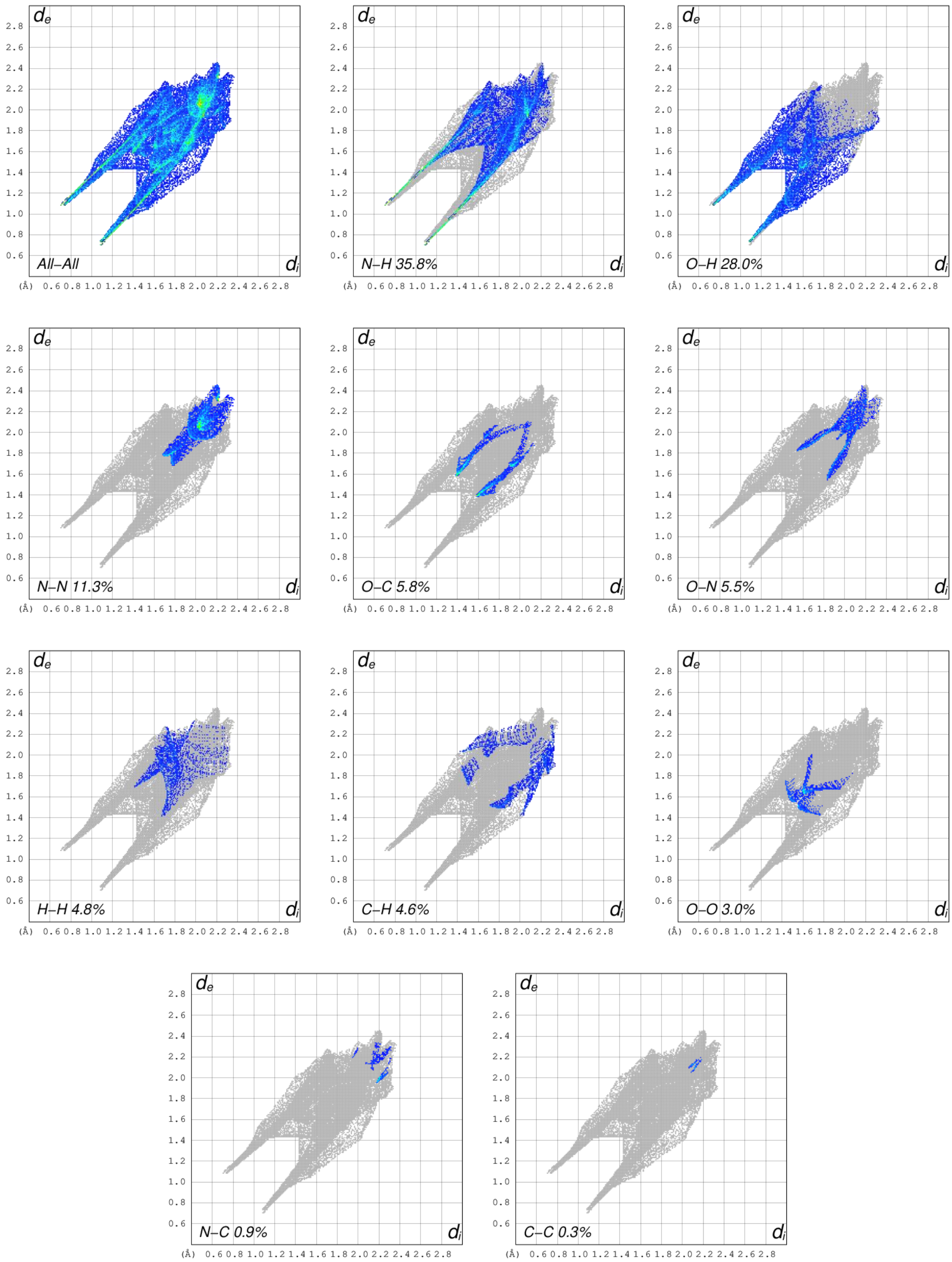

Figure S 37: Fingerprint plots for 2,2,3,3-tetrahydroxysuccinonitrile at 100 K mapped from the Hirshfeld surface. Surface contacts and overall quantity are provided in each graph.

6. Details on quantum chemical calculations

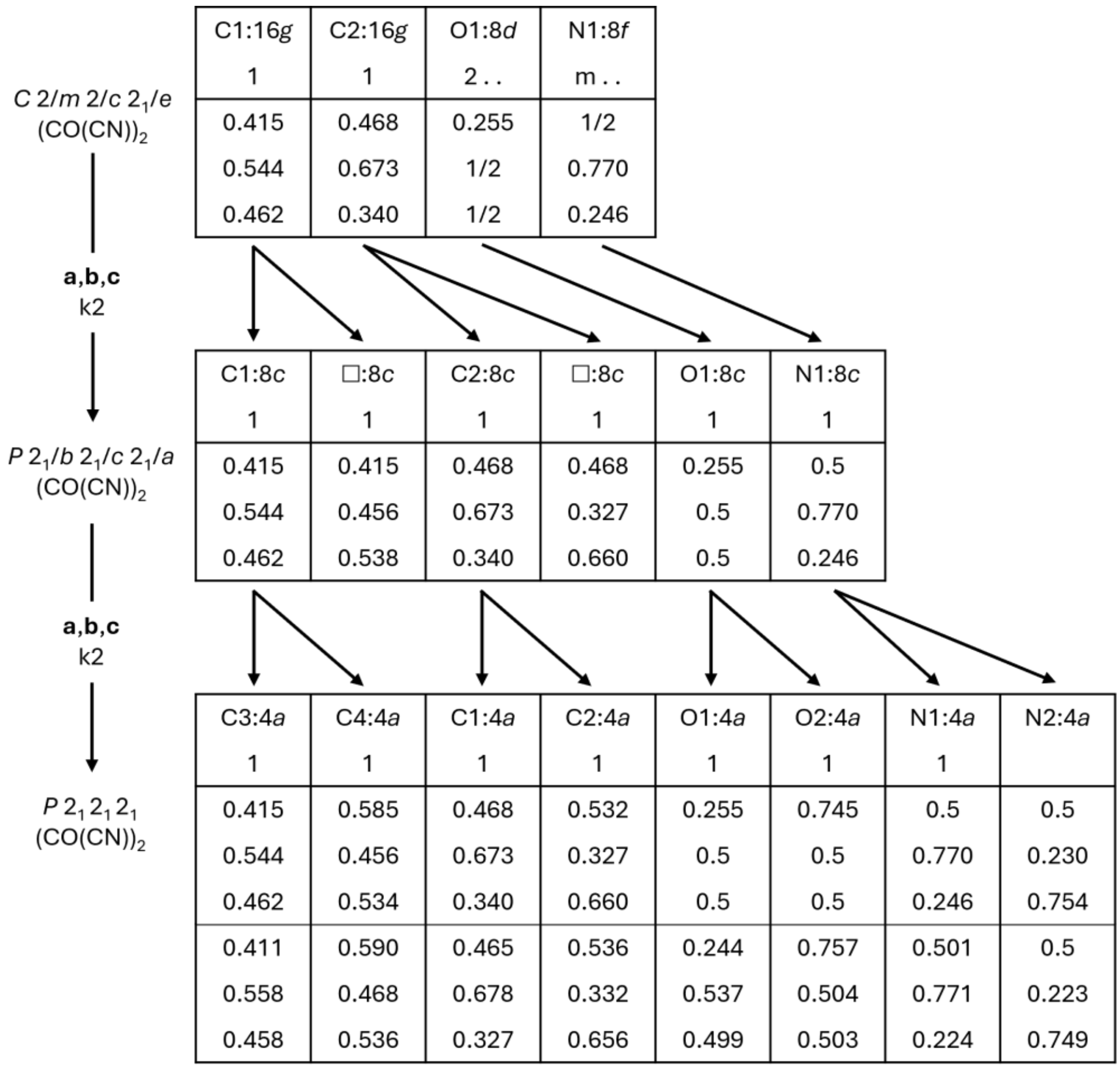

Figure S 38: Bärnighausen tree for the symmetry reduction of the disordered crystal structure of oxalyl dicyanide. Two *klassengleiche* symmetry reductions of index 2 lead to an ordered model with all atoms occupying the general Wyckoff position 4*a*. Optimized coordinates from quantum chemical calculations are given in the bottom row. Vacant positions of the disorder in $P2_12_12_1$ are not shown.

Table S 2: Lattice parameters and atomic coordinates of the optimized solid-state structure of oxalyl dicyanide at in space group $P2_12_12_1$ with the resolved disorder.

Lattice parameter	*a* [Å]	*b* [Å]	*c* [Å]
	6.74006940	7.05900570	8.74370881

Atom	*x*	*y*	*z*
O1	0.24444	0.53671	0.49935
O2	0.75691	0.50375	0.50267
N1	0.50069	0.77115	0.22354
N2	0.49984	0.22321	0.74876
C1	0.46464	0.67759	0.32687
C2	0.53619	0.33187	0.65558
C3	0.41091	0.55843	0.45786
C4	0.58999	0.46831	0.53639

Table S 3: Lattice parameters and atomic coordinates of the optimized solid-state structure of the 1,4-dioxane oxalyl dicyanide complex in space group $P\bar{1}$.

Lattice parameter	*a* [Å]	*b* [Å]	*c* [Å]
	5.64195625	6.59264540	6.86402704
	α [°]	*β* [°]	*γ* [°]
	107.524022	113.460116	97.4185510

Atom	*x*	*y*	*z*
O1	0.75388	0.63712	0.48820
O2	0.67735	0.19119	0.18725
C3	0.88760	0.51503	0.53282
N4	0.83698	0.28414	0.77080
C5	0.85971	0.38562	0.66636
C6	0.40530	0.18342	0.05740
H7	0.28473	0.13385	0.13564
H8	0.40250	0.35003	0.06329
C9	0.29789	0.02049	0.81258
H10	0.40947	0.07677	0.73085
H11	0.08455	0.00156	0.71449

Table S 4: Lattice parameters and atomic coordinates of the optimized solid-state structure of 2,2,3,3-tetrahydroxysuccinonitrile in space group $P2_1/n$.

Lattice parameter	*a* [Å]	*b* [Å]	*c* [Å]	*β* [°]
	6.90687830	6.10498935	7.19947628	111.746233

Atom	*x*	*y*	*z*
O1	0.43584	0.22907	–0.43973
O2	–0.32880	0.48700	–0.25176
N3	–0.18994	0.29133	0.37281
C4	–0.43484	0.40261	–0.43608
C5	–0.29471	0.33268	0.45924
H6	–0.23521	0.38156	–0.15782
H7	0.39878	0.23211	–0.31955

Table S 5: Comparison and band assignment of the calculated and experimental Raman spectrum (> 200 cm^{-1}) of oxalyl dicyanide. The irreducible representations are given for the point group of the respective space group $P2_12_12_1$ of the optimized crystal structure. Values in [cm^{-1}].

obs.	calc.	irrep.	assignment	obs.	calc.	irrep.	assignment
					744	*A*	
					745	B_3	
				712	751	B_1	C–C≡N in-phase bend
					752	B_2	
					801	B_1	
					801	B_2	asym C(3)–C(4) out-of-
	206	B_1	C≡N in-plane scissoring	771	802	*A*	plane
n.o.	215	B_3			803	B_3	
	231	B_2			954	B_1	
	241	B_1	sym. wagging whole		958	*A*	C(3)–C(1) + C(4)–C(3)
	250	B_3	molecule	n.o.	959	B_2	asym stretch
n.o.	260	B_2			971	B_3	
	262	*A*			1255	*A*	
	287	*A*	asym. wagging whole		1261	B_3	
	291	B_3	molecule	n.o.	1266	B_2	C(3)–C(4) stretch
261	292	B_1			1270	B_1	
	293	B_2		1731	1858	B_2	C=O stretch

261	301	*A*	C–C≡N in-plane and out-of-plane scissoring		1863	*A*	
	301	B_1			1870	B_3	
	305	B_3			1870	B_1	
	305	B_2			1872	*A*	
456	470	B_1	Skeletal deformation		1874	B_1	
	472	B_2			1876	B_2	
	473	*A*			1880	B_3	
	475	B_3		2245	2416	*A*	In phase & out of phase C≡N stretch
480	502	B_1	Skeletal deformation		2416	B_3	
	503	*A*			2416	B_1	
	503	B_2			2416	B_2	
	506	B_3			2417	B_1	
517	535	B_1	C(1)–C(3) –C(4)–C(2) wagging		2417	B_2	
	538	*A*			2418	B_3	
	539	B_3			2418	*A*	
	540	B_2					
n.o.	639	B_3	C–C≡N in-phase bend				
	640	*A*					
	643	B_2					
	644	B_1					

Table S 6: Comparison and band assignment of the calculated and experimental IR spectrum (> 450 cm^{-1}) of oxalyl dicyanide. The irreducible representations are given for the point group of the respective space group $P2_12_12_1$ of the optimized crystal structure. Values in [cm^{-1}].

obs.	calc.	irrep.	assignment	obs.	calc.	irrep.	assignment
n.o.	470	B_1	Skeletal deformation	922	954	B_1	C(3)–C(1) + C(4)–C(3) asym stretch
	472	B_2			959	B_2	
	475	B_3			971	B_3	
n.o.	502	B_1	Skeletal deformation	n.o.	1261	B_3	C(3)–C(4) stretch
	503	B_2			1266	B_2	
	506	B_3			1270	B_1	
487	535	B_1	C(1)–C(3) –C(4)–C(2) wagging	1738	1858	B_2	C=O stretch
	539	B_3			1870	B_3	
	540	B_2			1870	B_1	
612	639	B_3	C–C≡N in-phase bend		1874	B_1	
	643	B_2			1876	B_2	
	644	B_1			1880	B_3	
n.o.	745	B_3	C–C≡N in-phase bend	2250	2416	B_3	In phase & out of phase C≡N stretch
	751	B_1			2416	B_1	
	752	B_2			2416	B_2	
n.o.	801	B_1	asym C(3)–C(4) out-of-plane		2417	B_1	
	801	B_2			2417	B_2	
	803	B_3			2418	B_3	
